# Sequential stabilization of RNF220 by RLIM and ZC4H2 during cerebellum development and Shh-group medulloblastoma progression

**DOI:** 10.1093/jmcb/mjab082

**Published:** 2022-01-18

**Authors:** Yuwei Li, Chencheng Yang, Huishan Wang, Ling Zhao, Qinghua Kong, Yu Cang, Shuhua Zhao, Longbao Lv, Yan Li, Bingyu Mao, Pengcheng Ma

**Affiliations:** State Key Laboratory of Genetic Resources and Evolution, Kunming Institute of Zoology, Chinese Academy of Sciences, Kunming 650223, China; Kunming College of Life Science, University of Chinese Academy of Sciences, Kunming 650203, China; State Key Laboratory of Genetic Resources and Evolution, Kunming Institute of Zoology, Chinese Academy of Sciences, Kunming 650223, China; Kunming College of Life Science, University of Chinese Academy of Sciences, Kunming 650203, China; State Key Laboratory of Genetic Resources and Evolution, Kunming Institute of Zoology, Chinese Academy of Sciences, Kunming 650223, China; Kunming College of Life Science, University of Chinese Academy of Sciences, Kunming 650203, China; Experimental Animal Center, Kunming Institute of Zoology, Chinese Academy of Sciences, Kunming 650223, China; Kunming Institute of Botany, Chinese Academy of Sciences, Kunming 650223, China; Department of Urology, the Affiliated Hospital of Yunnan University, Kunming 650021, China; First Affiliated Hospital of Kunming Medical University, Kunming 650032, China; Experimental Animal Center, Kunming Institute of Zoology, Chinese Academy of Sciences, Kunming 650223, China; Kunming Institute of Botany, Chinese Academy of Sciences, Kunming 650223, China; State Key Laboratory of Genetic Resources and Evolution, Kunming Institute of Zoology, Chinese Academy of Sciences, Kunming 650223, China; Center for Excellence in Animal Evolution and Genetics, Chinese Academy of Sciences, Kunming 650223, China; State Key Laboratory of Genetic Resources and Evolution, Kunming Institute of Zoology, Chinese Academy of Sciences, Kunming 650223, China

**Keywords:** ZC4H2, RLIM, RNF220, Shh signaling, cerebellum, medulloblastoma (MB)

## Abstract

Sonic hedgehog (Shh) signaling is essential for the proliferation of cerebellar granule neuron progenitors (CGNPs), and its misregulation is linked to various disorders, including cerebellar cancer medulloblastoma (MB). During vertebrate neural development, RNF220, a ubiquitin E3 ligase, is involved in spinal cord patterning by modulating the subcellular location of glioma-associated oncogene homologs (Glis) through ubiquitination. RNF220 is also required for full activation of Shh signaling during cerebellum development in an epigenetic manner through targeting embryonic ectoderm development. ZC4H2 was reported to be involved in spinal cord patterning by acting as an RNF220 stabilizer. Here, we provided evidence to show that ZC4H2 is also required for full activation of Shh signaling in CGNP and MB progression by stabilizing RNF220. In addition, we found that the ubiquitin E3 ligase RING finger LIM domain-binding protein (RLIM) is responsible for ZC4H2 stabilization via direct ubiquitination, through which RNF220 is also thus stabilized. RLIM is a direct target of Shh signaling and is also required for full activation of Shh signaling in CGNP and MB cell proliferation. We further provided clinical evidence to show that the RLIM‒ZC4H2‒RNF220 cascade is involved in Shh-group MB progression. Disease-causative human RLIM and ZC4H2 mutations affect their interaction and regulation. Therefore, our study sheds light on the regulation of Shh signaling during cerebellar development and MB progression and provides insights into neural disorders caused by RLIM or ZC4H2 mutations.

## Introduction

The Sonic hedgehog (Shh) signaling pathway plays a key role in determining the fate of stem cells and body patterning during embryogenesis (McGlinn and Tabin, 2006; Ribes and Briscoe, 2009; Briscoe and Therond, 2013; Lopez-Rios, 2016). Its aberrant activation can lead to the development of multiple types of cancer; accordingly, targeting the Shh pathway is a promising therapeutic strategy for cancer treatment (Mullor et al., 2002; Pasca di Magliano and Hebrok, 2003). During normal cerebellar development, Shh acts as a mitogen and stimulates the proliferation of cerebellar granule neuron progenitors (CGNPs) (Fuccillo et al., 2006; Vaillant and Monard, 2009). CGNPs are specified starting at mouse embryonic day 13.5 and then undergo intense Shh-driven proliferation at the external granular layer (EGL) before becoming postmitotic and migrating inward to form the internal granular layer (IGL) of the mature cerebellum (Vaillant and Monard, 2009; Hatten and Roussel, 2011; De Luca et al., 2016). Mutations resulting in overactivation and/or deregulation of Shh signaling, including loss of patched 1 (Ptch1), alter the development of CGNP, making them hyperproliferative and susceptible to malignant transformation into medulloblastoma (MB), the most common malignant pediatric brain tumor (Goodrich et al., 1997; Briscoe and Therond, 2013; Marshall et al., 2014). The identification of four genetic subgroups of MB provides a rationale for studying molecular-based therapies in an effort to improve disease survival and reduce treatment-related side effects (Northcott et al., 2012, 2019; Garcia-Lopez et al., 2021). Moreover, as overactivated Shh signaling causes ∼30% MB, the biological and pathogenic importance of Shh signaling emphasizes the need to tightly control its action (Romer and Curran, 2005; Northcott et al., 2012).

The ligand-dependent Shh signaling cascade is initiated through ligand binding to the Shh receptor Ptch1, which abrogates the repression of the seven-pass transmembrane protein Smoothened (Smo). Smo then translocates into the cilium, driving a signaling cascade that culminates in the accumulation of glioma-associated oncogene (Gli) activators (Goetz and Anderson, 2010; Briscoe and Therond, 2013). These activator forms of Gli stimulate the transcription of a panel of target genes, including Gli1, Ptch1, and Hhip1, which also contribute to a feedback loop that regulates Shh signaling (Winklmayr et al., 2010; Hui and Angers, 2011).

We previously identified that the ubiquitin E3 ligase RNF220 was involved in ventral spinal cord patterning by promoting lysine 63 (K63)-linked polyubiquitination and nuclear exportation of Glis (Ma et al., 2019). RNF220 also acts as a Shh signaling enhancer via an epigenetic mechanism by targeting embryonic ectoderm development and is required for cerebellar development and Shh-group MB progression (Ma et al., 2020a). RNF220 mutations were recently reported to be associated with brain abnormalities, including leukodystrophy, ataxia, and sensorineural deafness (Sferra et al., 2021). ZC4H2, an X-linked gene, encodes a C4H2-type zinc-finger nuclear factor, whose mutations have been reported to be associated with various disorders, including arthrogryposis multiplex congeita, X-linked intellectual disability (XLID), Wieacker‒Wolff syndrome, and Miles‒Carpenter syndrome (Hirata et al., 2013; May et al., 2015; Zanzottera et al., 2017; Frints et al., 2019). Our previous studies showed that ZC4H2 is involved in neural induction by modulating bone morphogenetic protein/Smad signaling in *Xenopus* (Ma et al., 2017) and acts as a close interacting partner and stabilizer for RNF220, by which ZC4H2 is required for normal ventral spinal cord patterning (Ma et al., 2020b). In addition, the ubiquitin E3 ligase complex RNF220/ZC4H2 is also involved in the development of locus coeruleus (LC) by targeting the transcription factors Phox2a/b (Song et al., 2020). However, whether ZC4H2 plays a role during cerebellar development or MB progression and how the RNF220/ZC4H2 protein complex is regulated remain unknown.

The X-linked RLIM gene, also known as RNF12, encodes a widely expressed RING (really interesting new gene) domain-containing zinc-finger protein (Ostendorff et al., 2000). The RING finger LIM domain-binding protein (RLIM) has diverse functions. RLIM can both serve as a cofactor promoting or inhibiting the activity of LIM-homeodomain (LIM-HD) transcription factors and act as a ubiquitin E3 ligase ubiquitinating its target proteins for subsequent degradation by the proteasome, by which RLIM is involved in many biological processes, including early embryogenesis, X chromosome inactivation, cell migration, and proliferation (Bach et al., 1999; Hiratani et al., 2003; Huang et al., 2011; Gontan et al., 2012; Zhang et al., 2012; Gao et al., 2016). In humans, RLIM mutations were recently identified with XLID by inactivating the catalytic activity of ubiquitin E3 ligase (Tonne et al., 2015; Bustos et al., 2018; Frints et al., 2018; Palmer et al., 2020).

Here, we report that ZC4H2 is required for full activation of Shh signaling in CGNP and MB cell proliferation by stabilizing RNF220. We found that RLIM mediates K11- and K63-linked polyubiquitination of ZC4H2, by which ZC4H2 is stabilized. We also show that RLIM is a direct target of Shh/Gli signaling and is required for full activation of Shh signaling in CGNP and MB cell proliferation. In addition, we provide evidence that the RLIM‒ZC4H2‒RNF220 cascade is involved in MB progression in a mouse model as well as in clinical samples. Furthermore, we provide evidence to suggest that impaired RLIM‒ZC4H2 interaction and regulation caused by disease-causative mutations might contribute to the related disease progression.

## Results

### ZC4H2 is required for CGNP proliferation through RNF220

**Figure 1:**
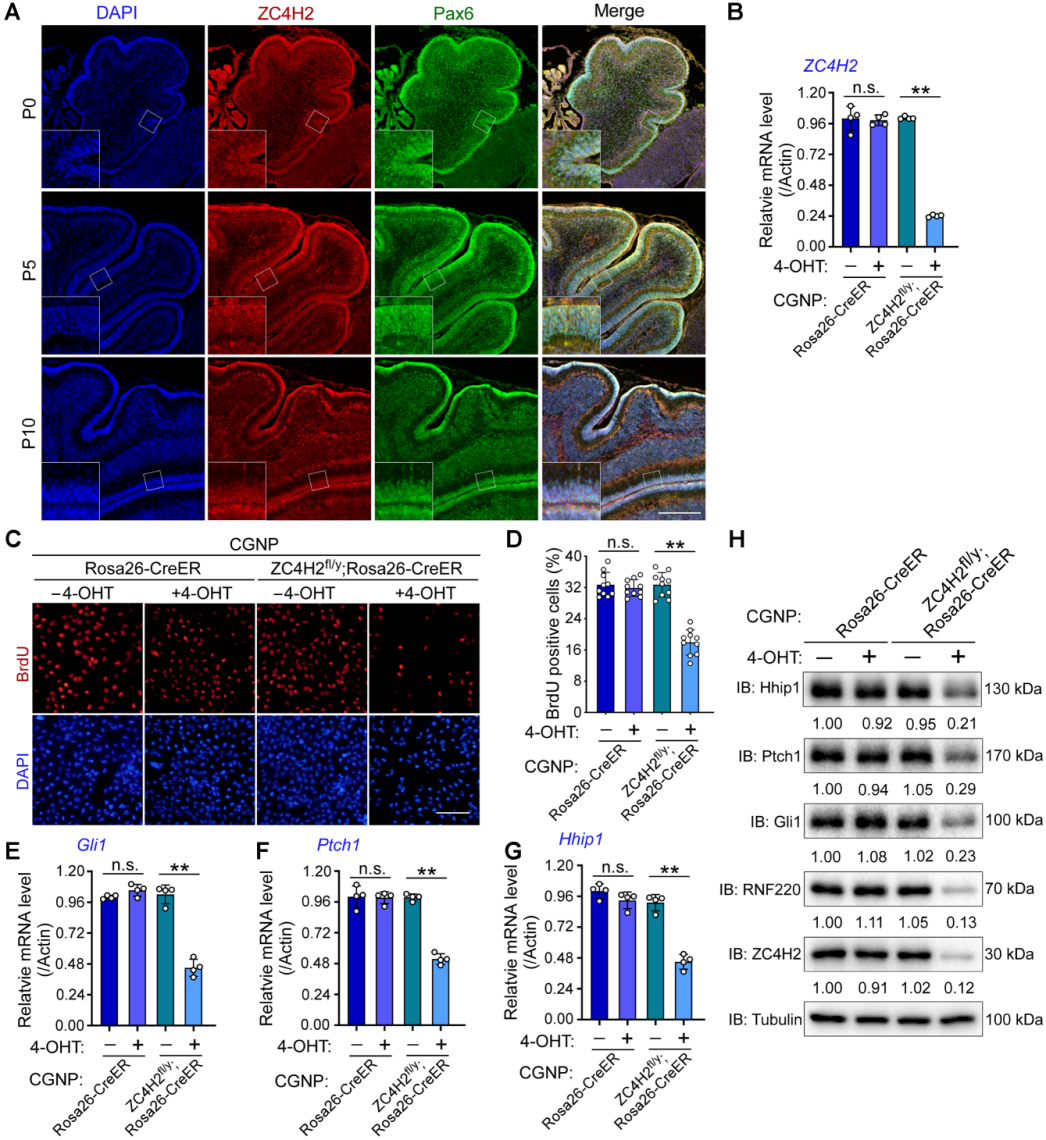
ZC4H2 is required for proliferation and Shh signaling in CGNPs. (**A**) ZC4H2 (red) and Pax6 (green) costaining immunofluorescence assay in cerebellum at different developmental stages. Scale bar, 200 µm. (**B**) RT-PCR assays showing the relative expression level of ZC4H2 in CGNPs from the indicated mouse. 4-OHT was used to induce ZC4H2 knockout. β-Actin was used as a loading control. (**C** and **D**) BrdU incorporation assay to evaluate DNA synthesis and proliferation rates of the indicated CGNPs treated with or without 4-OHT (**C**) and quantification of BrdU assay results (**D**). Scale bar, 50 µm. (**E**‒**H**) RT-PCR (**E**‒**G**) and western blotting (**H**) assays showing the expression levels of Gli1 (**E** and **H**), Ptch1 (**F** and **H**), and Hhip1 (**G** and **H**) in the indicated CGNPs treated with or without 4-OHT. The protein levels were normalized against α-tubulin and the statistics were labelled below each blot panel. **P < 0.01 (Student's *t*-test). IB, immunoblot; n.s., no significant difference.

We previously reported that RNF220, a ZC4H2 partner and target, is expressed at the EGL during cerebellar development (Ma et al., 2020a, b). Here, we first examined the expression of ZC4H2 during mouse cerebellar development and found that ZC4H2 was specifically expressed in proliferating CGNPs at the EGL of the developing cerebellum and colocalized with BrdU and the CGNP marker Pax6 (Figure 1A; Supplementary Figure S1A). To study the potential role of ZC4H2 in CGNP proliferation, we isolated CGNPs from the postnatal day 7 (P7) cerebellum of *Rosa26-CreER* and *ZC4H2^fl/^^y^;Rosa26-CreER* mice and examined the effect of ZC4H2 deletion induced by 4-hydroxytamoxifen (4-OHT) treatment on CGNP proliferation. We first stained primary CGNPs with Pax6 and found that nearly all isolated cells were Pax6-positive (Supplementary Figure S1B). 4-OHT treatment for 3 days efficiently decreased ZC4H2 expression in *ZC4H2^fl/^^y^;Rosa26-CreER* CGNPs (Figure 1B) and significantly decreased the bromodeoxyuridine (BrdU) incorporation ratio of *ZC4H2^fl/y^;Rosa26-CreER* CGNPs (Figure 1C and D). As CGNP proliferation is mainly driven by Shh signaling during cerebellar development (Vaillant and Monard, 2009), we next examined the expression levels of Shh targets in ZC4H2 knockout CGNPs. 4-OHT-mediated ZC4H2 knockout in *ZC4H2^fl/y^;Rosa26-CreER* CGNPs reduced both the mRNA and protein levels of Gli1, Ptch1, and Hhip1, three established Shh signaling targets (Figure 1E‒H; Winklmayr et al., 2010). These results suggest that ZC4H2 is required for the proliferation of CGNPs by modulating Shh signaling during cerebellar development.

### ZC4H2 contributes to Shh-group MB progression

Aberrant or sustained Shh signaling in CGNPs usually results in MB occurrence, and we previously proved the contribution of RNF220 during Shh-group MB progression (Ma et al., 2020a). Here, we first performed Dox-induced short hairpin RNA (shRNA)-mediated ZC4H2 knockdown experiments in Daoy cells, an MB cell line classified as the Shh subtype (Ivanov et al., 2016). The shRNA knockdown efficiency on ZC4H2 expression was demonstrated by real-time polymerase chain reaction (RT-PCR) assays (Supplementary Figure S2A). Cell proliferation was inhibited by ZC4H2 knockdown, as evidenced by the growth curve (Figure 2A) and BrdU incorporation analysis (Figure 2B and C). To test the role of ZC4H2 in MB progression, we injected Daoy cells stably transfected with inducible ZC4H2 shRNA into mice and induced ZC4H2 knockdown with Dox. Dox treatment led to decreased tumor size *in vivo* (Figure 2D). In addition, Dox-induced ZC4H2 knockdown significantly reduced Gli1, Ptch1, and Hhip1 mRNA and protein levels, as demonstrated by RT-PCR and western blotting results in Daoy cells (Figure 2E‒H).

**Figure 2:**
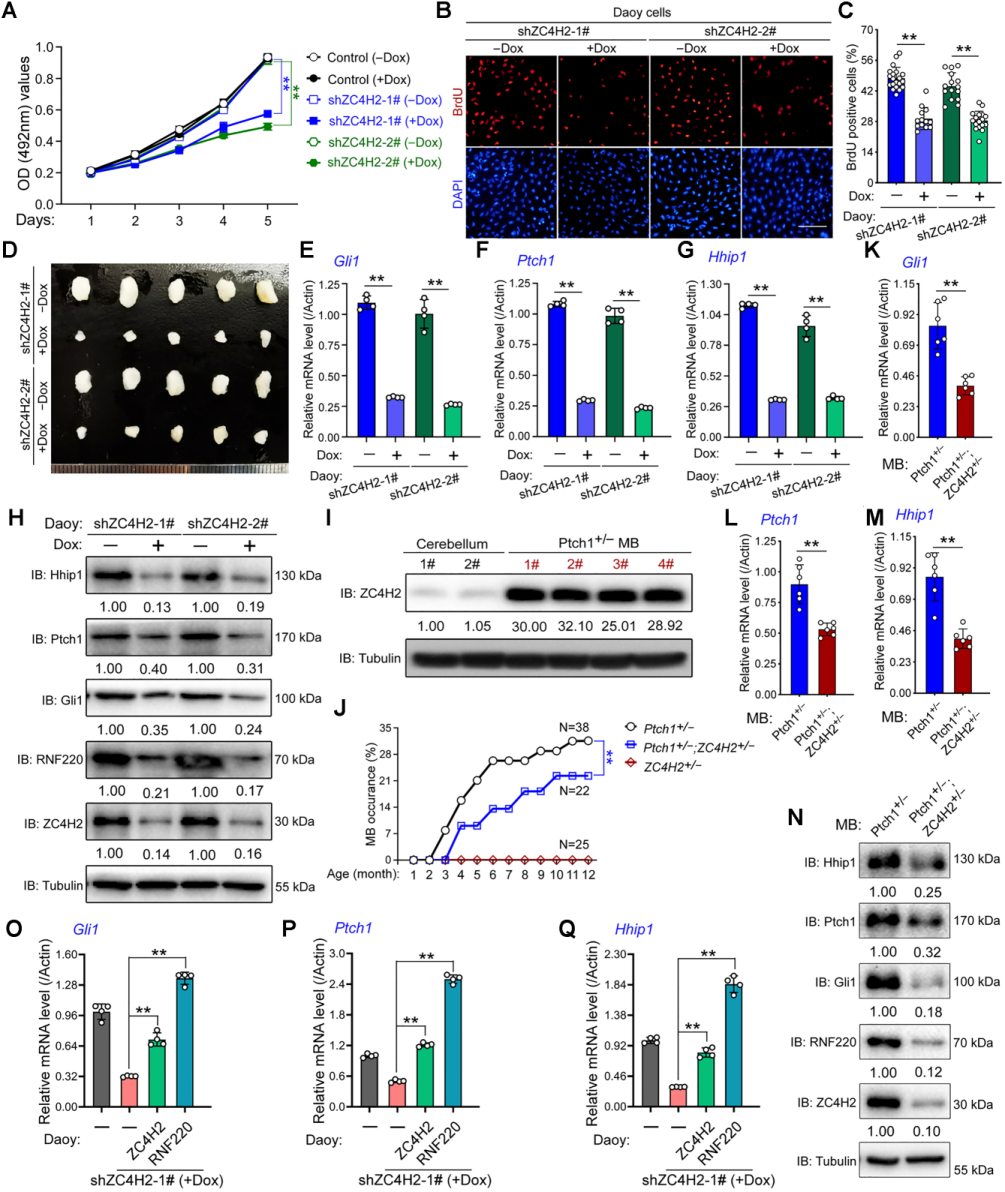
ZC4H2 is required for Daoy cell proliferation and xenograft growth. (**A**) Growth curve for control and ZC4H2 knockdown Daoy cell lines. Dox was used to induce ZC4H2 knockdown in shZC4H2-1# and shZC4H2-2# stable cell lines. **P < 0.01 (two-way ANOVA). (**B** and **C**) BrdU incorporation assay to evaluate DNA synthesis and proliferation rates of Daoy cells treated with or without Dox. Dox was used to induce ZC4H2 knockdown. Scale bar, 50 µm. **P < 0.01 (Student's *t*-test). (**D**) Photographs of xenograft tumors from BALB/c nude mice subcutaneously injected with shZC4H2-1# or shZC4H2-2# clone fed with or without Dox at 53 days after injection. (**E**‒**H**) RT-PCR and western blotting assays showing the expression level of Gli1 (**E** and **H**), Ptch1 (**F** and **H**), and Hhip1 (**G** and **H**) in the indicated Daoy cells treated

**Figure 2:**
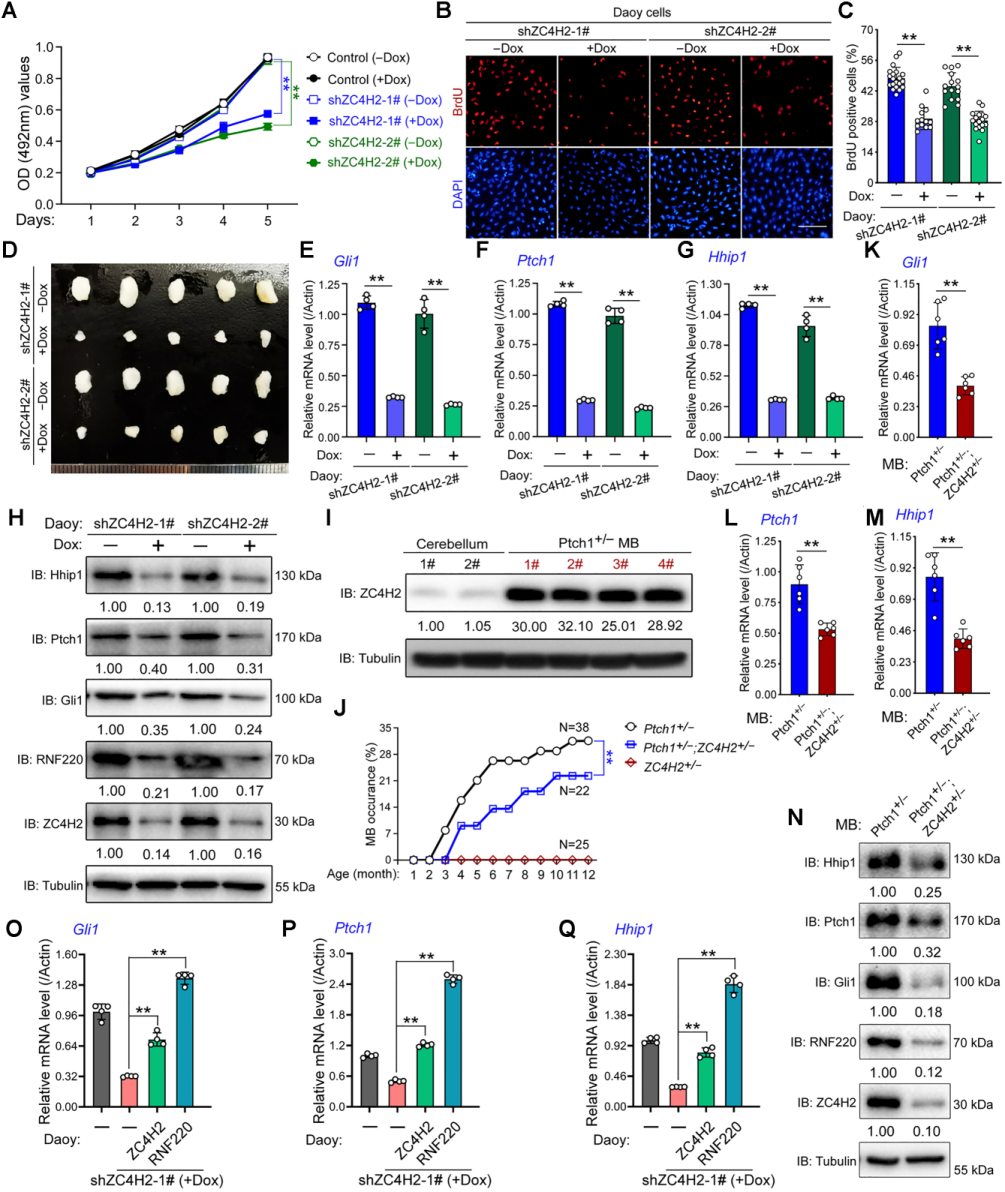
*(Continued)* with or without Dox. β-Actin was used as a loading control for RT-PCR assays. The protein levels were normalized against α-tubulin and the statistics were labelled below each blot panel. **P < 0.01 (Student's *t*-test). (**I**) Western blotting assays showing the protein level of ZC4H2 in control cerebellum and *Ptch1^+/−^* MB tissues. ZC4H2 protein levels were normalized against α-tubulin and the statistics were labelled below the blot panel. (**J**) Cumulative MB occurrence of the indicated mice during 12-month observation. **P < 0.01 (two-way ANOVA). (**K**‒**N**) RT-PCR (**K**‒**M**) and western blotting (**N**) assays showing the expression levels of Gli1 (**K** and **N**), Ptch1 (**L** and **N**), and Hhip1 (**M** and **N**) in MB tissue from the indicated mouse. β-Actin was used as a loading control for RT-PCR assays. The protein levels were normalized against α-tubulin and the statistics were labelled below each blot panel. **P < 0.01 (Student's *t*-test). (**O**‒**Q**) RT-PCR assays showing the expression level of Gli1 (**O**), Ptch1 (**P**), and Hhip1 (**Q**) in ZC4H2 knockdown Daoy cells coexpressed with shRNA-resistant ZC4H2 or RNF220. β-Actin was used as a loading control. **P < 0.01 (Student's *t*-test).

To further explore the involvement of ZC4H2 in MB genesis, we used a spontaneous orthotopic MB model driven by aberrant Shh signaling in *Ptch1^+/−^* mice (Goodrich et al., 1997). Although ZC4H2 mRNA levels were decreased in *Ptch1^+/−^* MB tissues (Supplementary Figure S2B), the ZC4H2 protein expression was markedly upregulated (Figure 2I), which suggests that ZC4H2 is stabilized at a posttranslational level during MB progression. We examined MB occurrence in *Ptch1^+/−^* and *ZC4H2^+/−^;Ptch1^+/−^* mice during the first 12 months after birth. *ZC4H2^+/−^;Ptch1^+/−^* mice were less prone to MB than *Ptch1^+/−^* mice, whereas the tested *ZC4H2^+/−^* mice showed absolutely no MB occurrence for the whole year (Figure 2J). We compared the expression levels of Gli1, Ptch1, and Hhip1 in different MB tissues using RT-PCR and western blotting assays, and the results showed that all three genes were downregulated in *ZC4H2^+/−^;Ptch1^+/−^* MB tissues compared with *Ptch1^+/−^* ones (Figure 2K–N).

We previously reported that ZC4H2 is required for RNF220 stabilization (Ma et al., 2020b). Here, the RNF220 protein level was indeed decreased in certain cases, including 4-OHT-induced ZC4H2 knockout in CGNPs (Figure 1H), Dox-induced ZC4H2 knockdown in Daoy cells (Figure 2H), and *ZC4H2^+/−^;Ptch1^+/−^* MB tissues (Figure 2N). Next, we asked whether the regulation of Shh signaling by ZC4H2 is RNF220-dependent. We examined the rescue activity of RNF220 expression in ZC4H2 knockdown Daoy cells on the expression level of Shh targets. The results showed that mRNA levels of Gli1, Ptch1, and Hhip1 could be rescued by either shRNA-resistant ZC4H2 or RNF220 coexpression (Figure 2O–Q), implying that ZC4H2-mediated RNF220 stabilization is involved in the regulation of ZC4H2 on Shh signaling in Daoy cells.

### ZC4H2 interacts with the E3 ligase RLIM

**Figure 3:**
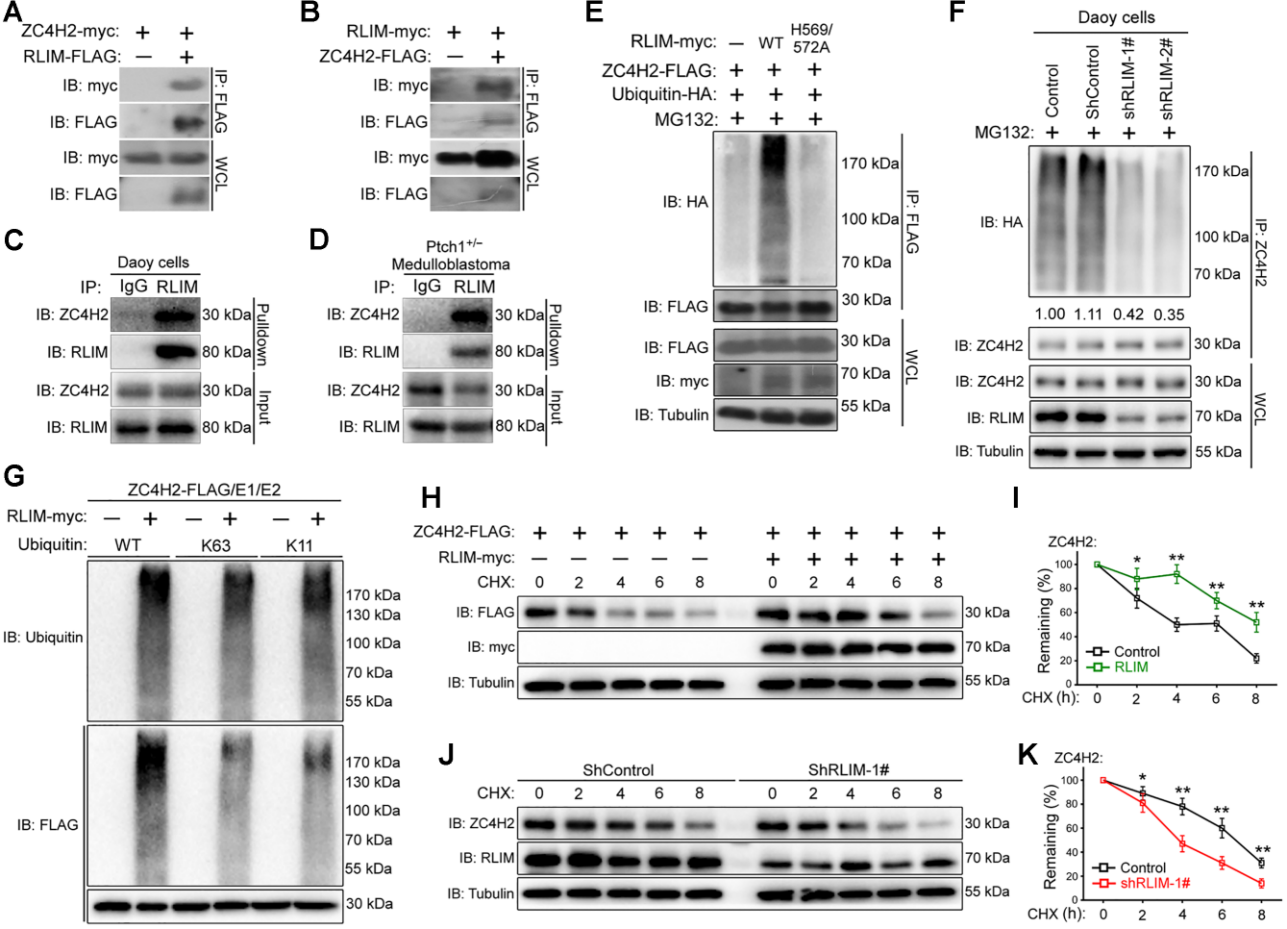
RLIM interacts with and targets ZC4H2 for polyubiquitination. (**A** and **B**) Co-IP assays showing the interaction between RLIM and ZC4H2 proteins. HEK293 cells were transiently transfected with different combinations of FLAG- or myc-tagged RLIM and ZC4H2 expression vectors as indicated. Cell lysates were incubated with anti-FLAG beads, washed, and subsequently analyzed by western blotting. (**C** and **D**) Endogenous ZC4H2 was pulled down by RLIM in both Daoy cells (**C**) and *Ptch1^+/−^* MB tissues (**D**). (**E**) *In vivo* polyubiquitination assays showing the polyubiquitinated ZC4H2 protein level when RLIM WT or the ligase-dead H569/572A mutant (both histidine 569 and histidine 572 mutated to alanine) was coexpressed in HEK293 cells. (**F**) The polyubiquitination level of endogenous ZC4H2 reduced when RLIM was knocked down in Daoy cells. The levels of polyubiquitinated ZC4H2 were quantified against α-tubulin followed by each control and the statistics are shown below the indicated panel. (**G**) *In vitro* ubiquitination assays showing ZC4H2 as a direct target of RLIM for K11 and K63 polyubiquitination. (**H**‒**K**) Effects of RLIM overexpression on the stability of ZC4H2. HEK293 cells were transiently transfected with the indicated plasmids (**H** and **I**), and shRNA stably transfected Daoy cells were plated in six-well plates (**J** and **K**). After 48 h, cycloheximide was added to all samples, and the cells were then harvested at the indicated time points. ZC4H2 protein levels were determined by western blotting with anti-FLAG or ZC4H2 antibody. In all cases, tubulin was used as a loading control. (**I** and **K**) The relative levels of ZC4H2 were quantified densitometrically and normalized against tubulin. Data shown are mean ± SD of three independent experiments. *P < 0.05 and **P < 0.01 (Student's *t*-test). WCL, whole-cell lysate; CHX, cycloheximide.

The above data suggest that ZC4H2 protein is stabilized at a posttranslational level during MB progression. To screen for its potential regulator, we used a yeast two-hybrid system and isolated one positive clone that encodes a partial sequence of the ubiquitin E3 ligase RLIM (Supplementary Figure S3A; Ma et al., 2020b). To confirm their interaction, we used co-immunoprecipitation (co-IP) assays. In HEK293 cells transiently cotransfected with FLAG-tagged RLIM and myc-tagged ZC4H2, ZC4H2 coimmunoprecipitated with RLIM (Figure 3A). In the reverse experiment, RLIM also coimmunoprecipitated with ZC4H2 (Figure 3B). When endogenous RLIM was immunoprecipitated from Daoy cell or the *Ptch1^+/−^* MB tissue lysate using an RLIM antibody, endogenous ZC4H2 was also detected in the immunoprecipitates (Figure 3C and D).

To map the domains of RLIM that are responsible for the observed interaction, a series of RLIM truncates were constructed and tested via co-IP assays in HEK293 cells. RLIM contains a characteristic RING finger domain at its C-terminus and a nuclear location signal (NLS) domain at the middle of the protein. We constructed ΔRING (with the RING finger domain deleted), ΔN (with the N-terminal amino acids 1‒200 deleted), ΔN/NLS (with the N-terminal amino acids 1‒300 deleted), and ΔN/RING (with the N-terminal amino acids 1‒200 and the RING domain deleted) (Supplementary Figure S3B). Our results showed that only the ΔN construct of RLIM could associate with ZC4H2 with comparable efficiency to the full-length RLIM protein, while ΔRING, ΔN/NLS, and ΔN/RING could not (Supplementary Figure S3C). The results suggest that RLIM nuclear localization and its RING domain are required for ZC4H2 interaction. RLIM is a cytoplasmic and nuclear shuttling protein, and the process is regulated by phosphorylation at the serine 214 (S214) site (Jiao et al., 2013). Next, we examined whether the nuclear localization of RLIM is required for ZC4H2 interaction by testing ΔNLS (with only the NLS domain deleted) and S214A mutation of RLIM in co-IP experiments. We first checked the subcellular localization of these constructs and found that ΔNLS and S214A mutation of RLIM wild-type (WT) or RLIM ΔR translocated from the nucleus to the cytoplasm (Supplementary Figure S3D). Co-IP results showed that neither ΔNLS nor S214A mutation of RLIM WT and RLIM ΔR could be associated with ZC4H2 (Supplementary Figure S3E and F). These results suggest that the RLIM‒ZC4H2 interaction occurs in the nucleus in a RING domain-dependent manner.

### RLIM polyubiquitinates and stabilizes ZC4H2

Given that RLIM is an E3 ubiquitin ligase that often regulates the stability of its substrates and that ZC4H2 could stabilize its interacting partner through reduced polyubiquitination, we first examined whether their protein levels are regulated by each other (Gontan et al., 2012; Ma et al., 2020b). Unexpectedly, cotransfection of RLIM WT but not its ligase-dead mutant (H569/572A, both histidine 569 and histidine 572 mutated to alanine) stabilized ZC4H2 protein in HEK293 cells, while the protein levels of neither RLIM WT nor RLIM H569/572A were affected by ZC4H2 coexpression (Supplementary Figure S4A and B; Bustos et al., 2018). We further carried out ubiquitination assays to examine whether RLIM may affect the ubiquitination status of ZC4H2 and found that coexpression of RLIM promotes the polyubiquitination of ZC4H2, and this activity was largely eliminated when its ligase-dead mutant was used (Figure 3E).

Ubiquitin ligase usually promotes the degradation of target proteins, but RLIM coexpression in fact stabilizes ZC4H2. Ubiquitin ligases can add different types of ubiquitin chains to their substrates, with different functional effects. K48-linked polyubiquitin chains target proteins for destruction, and K63- or K11-linked ubiquitin chains have mostly been implicated in nonproteolytic regulation (Husnjak and Dikic, 2012). To characterize the type of ubiquitin chains added by RLIM to ZC4H2, different ubiquitin mutants were used in ubiquitination assays, in which either all lysines of ubiquitin (KO) or all lysines except K6, K11, K27, K29, K33, K48, and K63 (K6/11/27/29/33/48/63) are substituted by arginines or K11 and K63 are substituted by arginine separately (K11R, K63R) or together (K11/63R). The results showed that RLIM promoted the mixed K11- and K63-linked polyubiquitination of ZC4H2, consistent with the nonproteolytic nature of the modification (Supplementary Figure S4C and D).

We examined the levels of polyubiquitinated ZC4H2 in Daoy cells transfected with control or RLIM shRNAs, which were found to be reduced by ∼60% in RLIM knockdown cells (Figure 3F), supporting the notion that RLIM plays a role in ZC4H2 polyubiquitination *in vivo*. To prove that RLIM directly ubiquitinates ZC4H2, *in vitro* ubiquitination assays were carried out using purified RLIM and ZC4H2 proteins and specific ubiquitin mutants. RLIM efficiently promoted K11- and K63-linked ubiquitination of ZC4H2 *in vitro* (Figure 3G).

To further confirm the ZC4H2-stabilizing activity of RLIM, we used a series of cycloheximide-based protein chases to test the protein stability of ZC4H2 when RLIM was overexpressed or knocked down. In the presence of RLIM, ZC4H2 was clearly stabilized in HEK293 cells, while RLIM knockdown in Daoy cells reduced ZC4H2 stability (Figure 3H‒K). Note that neither the RNF220 nor the ZC4H2 mRNA level was affected by RLIM overexpression or knockdown (Supplementary Figure S5).

### RLIM is a direct target of Shh/Gli signaling

**Figure 4:**
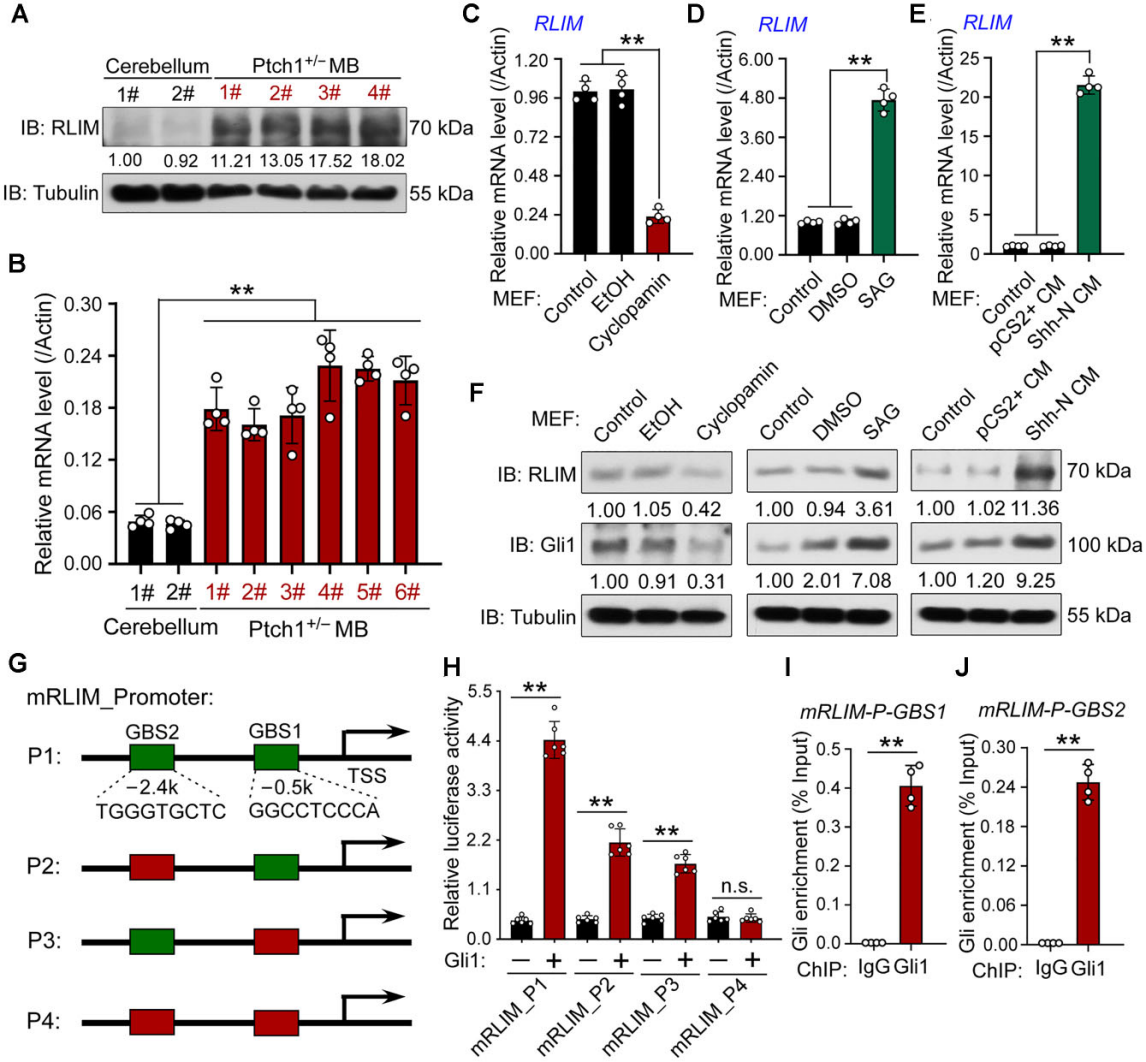
RLIM is a direct target of Shh signaling. (**A** and **B**) Western blotting (**A**) and RT-PCR (**B**) assays showing RLIM expression levels in control cerebellum and Ptch1^+/^*^−^* MB tissues. RLIM protein levels were normalized against α-tubulin and the statistics are shown below the blot panel. β-Actin was used as a loading control for RT-PCR assays. (**C**‒**F**) RT-PCR (**C**‒**E**) and western blotting (**F**) assays showing RLIM levels in MEF cells treated with the indicated chemicals (**C, D**, and **F**) or conditioned medium (**E** and **F**). β-Actin was used as a loading control for RT-PCR assays. The protein levels were normalized against α-tubulin and the statistics were labelled below each blot panel. (**G**) Schematic presentation of luciferase reporter vectors of mouse RLIM promoters containing the two predicted wild-type (green) or mutated (red) potential GBSs used in the reporter assays. (**H**) Luciferase reporter assays showing two functional GBSs in mouse RLIM promoter. RLUs, relative luciferase units. (**I** and **J**) ChIP‒qPCR assays showing that Gli1 directly binds to mouse RLIM promoter. **P < 0.01 (Student's *t*-test).

Next, we examined RLIM expression in the control cerebellum and *Ptch1^+/^*^–^ MB tissues and found that both RLIM mRNA and protein levels were upregulated in *Ptch1^+/^*^–^ MB tissues (Figure 4A and B), which implies that RLIM is a potential target of Shh signaling. To test this hypothesis, we examined the RLIM level when Shh signaling was modulated by various methods, including Shh-conditioned medium (Shh-CM), recombinant Shh protein, Smo agonist (SAG), and inhibitors (cyclopamine, GDC-0449/vismodegib, and LDE225/sonidegib), in CGNP and MEF cells. The results showed that both RLIM mRNA and protein levels were upregulated when Shh signaling was activated but downregulated when Shh signaling was inhibited (Figure 4C‒F; Supplementary Figure S6).

By searching the mouse and human RLIM promoters, we found two and three potential Gli-binding sequences (GBSs), respectively, with seven matches of the common nine bases (GACCACCCA) (Figure 4G; Supplementary Figure S7A; Winklmayr et al., 2010). Reporter gene assays showed that Gli activator coexpression activated reporter gene expression driven by the RLIM promoter containing the potential GBS. Mutation of mouse GBS1/GBS2 or human GBS1/GBS3 attenuated the effect of Gli on RLIM promoter activity, and double mutation of these two sites almost abolished the activity (Figure 4H; Supplementary Figure S7B). Next, we performed chromatin immunoprecipitation‒quantitative PCR (ChIP‒qPCR) experiments to test whether these GBSs in the RLIM promoter recruit Gli, and the results showed that genome sequences covering mouse GBS1/2 or human GBS1/3 sites accumulated in Gli immunoprecipitates in SAG-treated NIH3T3 or Daoy cells (Figure 4I and J; Supplementary Figure S7C and D). Taken together, these results suggest that Shh signaling activates RLIM expression through the binding of Gli to the RLIM promoter.

### RLIM is required for full activation of Shh signaling through ZC4H2/RNF220

**Figure 5:**
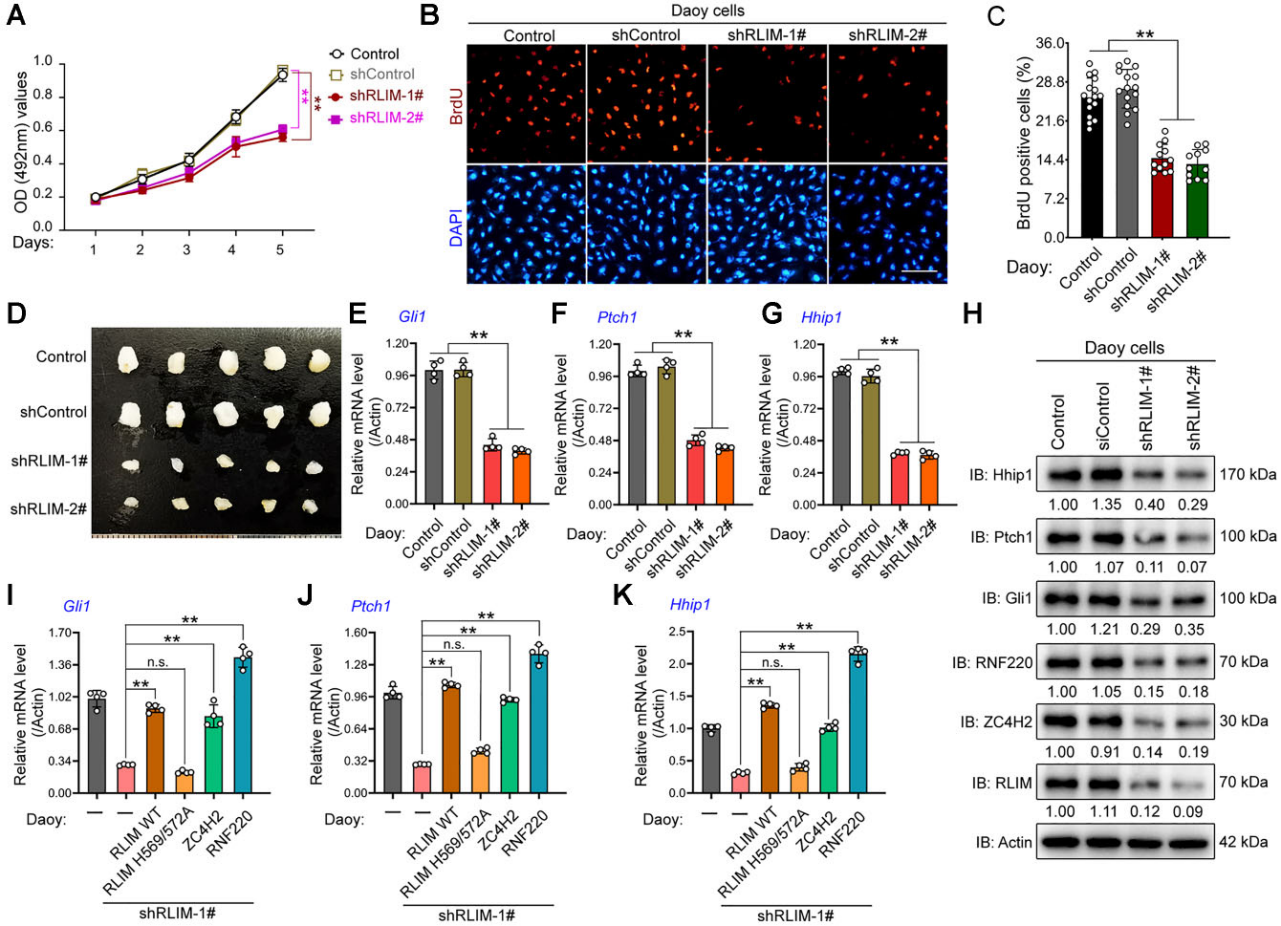
RLIM is required for Daoy cell proliferation and xenograft growth. (**A**) Growth curve for control and RLIM knockdown Daoy cell lines. **P < 0.01 (two-way ANOVA). (**B** and **C**) BrdU incorporation assay to evaluate DNA synthesis and proliferation rates of control or shRLIM stably transfected Daoy cells (**B**) and quantification of BrdU assay results (**C**). Scale bar, 50 µm. **P < 0.01 (Student's *t*-test). (**D**) Photographs of xenograft tumors from BALB/c nude mice subcutaneously injected with shRLIM-1# or hRLIM-2# clone at 53 days after injection. (**E**‒**H**) RT-PCR (**E**‒**G**) and western blotting (**H**) assays showing the expression levels of Gli1 (**E** and **H**), Ptch1 (**F** and **H**), and Hhip1 (**G** and **H**) in the indicated Daoy cells. β-Actin was used as a loading control for RT-PCR assays. The protein levels were normalized against α-tubulin and the statistics were labelled below each blot panel. **P < 0.01 (Student's *t*-test). IB, immunoblot. (**I**‒**K**) RT-PCR assays showing the expression level of Gli1 (**I**), Ptch1 (**J**), and Hhip1 (**K**) in RLIM knockdown Daoy cells coexpressed with shRNA-resistant wild-type (WT) or ligase-dead (H569/572A) RLIM, ZC4H2, or RNF220. **P < 0.01 (Student's *t*-test). n.s., not significant difference.

As RLIM is overexpressed in *Ptch1^+/^*^–^ MB tissues, we examined the effect of RLIM on MB progression in Daoy cells via shRNA-mediated knockdown assays. The shRNA knockdown efficiency on RLIM expression was demonstrated by RT-PCR (Supplementary Figure S8A). Cell proliferation was inhibited by RLIM knockdown, as evidenced by the growth curve and BrdU incorporation analysis (Figure 5A‒C). To test the role of RLIM in MB progression, we injected Daoy cells stably transfected with RLIM shRNA into mice and found that RLIM knockdown led to reduced tumor size *in vivo* (Figure 5D). In addition, RLIM knockdown significantly reduced Gli1, Ptch1, and Hhip1 at both mRNA and protein levels in Daoy cells (Figure 5E‒H).

ZC4H2 and RNF220 protein expression was indeed decreased in RLIM knockdown cells (Figure 5H). We conducted a series of rescue experiments by coexpression of shRNA-resistant RLIM, ZC4H2, or RNF220 in RLIM shRNA stably transfected cells. Indeed, we found that the expression of Gli1, Ptch1, and Hhip1 was rescued by coexpression of shRNA-resistant RLIM WT but not its ligase-dead mutant in RLIM knockdown cells (Figure 5I‒K). Additionally, the expression of Gli1, Ptch1, and Hhip1 was derepressed by coexpression of either ZC4H2 or RNF220 in RLIM knockdown cells (Figure 5I‒K).

A previous study reported that RLIM was expressed in Purkinje and granule progenitor cells in the developing rat cerebellum (Hayes et al., 2001). Here, we provided evidence showing that RLIM was expressed in proliferating CGNPs at the EGL during cerebellar development in mice and colocalized with BrdU and Pax6 (Supplementary Figure S9). To prove the potential role of RLIM during Shh-driven granule progenitor cell proliferation, shRNAs were used to knock down RLIM in CGNPs. Indeed, two independent RLIM shRNAs worked efficiently to knock down endogenous RLIM in CGNPs, as evidenced by RT-PCR (Supplementary Figure S8B). BrdU incorporation analysis showed that RLIM knockdown in CGNPs inhibited cell proliferation (Supplementary Figure S8C and D). Next, we examined the expression of Gli1, Ptch1, and Hhip1 in CGNPs when RLIM was knocked down by shRNAs. Additionally, mRNA and protein levels of Gli1, Ptch1, and Hhip1 were decreased in RLIM knockdown CGNPs (Supplementary Figure S8E‒H). Note that protein levels of both ZC4H2 and RNF220 were decreased in RLIM knockdown CGNPs (Supplementary Figure S8H).

### The RLIM‒ZC4H2‒RNF220 axis is involved in Shh-group MB progression

**Figure 6:**
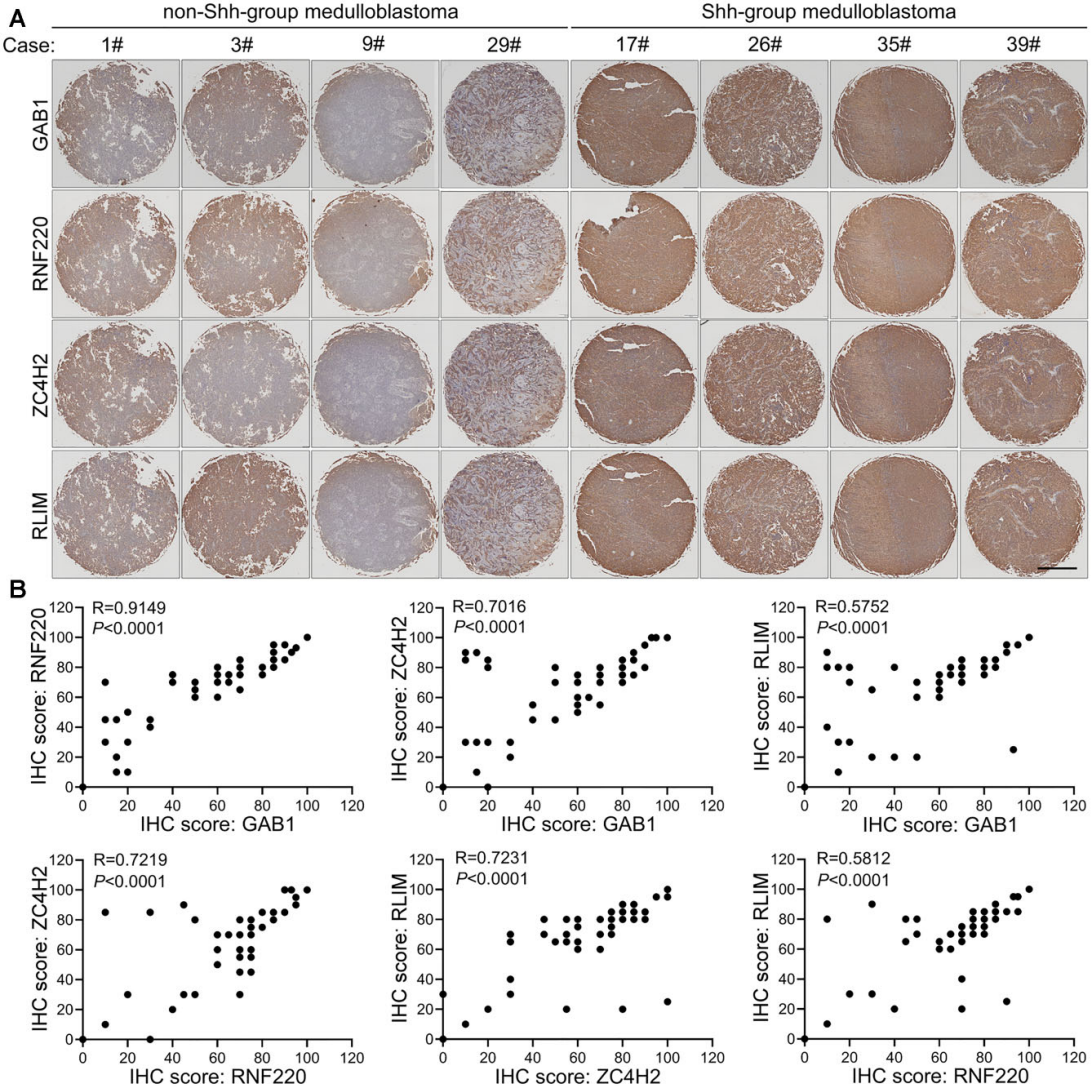
Expression correlation analysis among RLIM, ZC4H2, and RNF220 in clinical MB tissues. (**A**) Representative immunohistochemical images of clinical Shh (left four panels) and non-Shh (right four panels) MB samples with the indicated antibodies. Scale bar, 5 mm. (**B**) Statistical analysis of the correlation among GAB1, RNF220, ZC4H2, and RLIM based on the immunohistochemical staining scores. Pearson product-moment correlation coefficient analysis was used for the statistics.

To examine the potential clinical association between ZC4H2/RNF220 and RLIM, we detected the expression of ZC4H2/RNF220 and RLIM in 39 human MB specimens. Shh-group MB was identified by GRB2-associated binding protein 1 (GAB1) staining (Kaur et al., 2016). As previously reported, RNF220 expression correlated well with that of GAB1, confirming the role of RNF220 during Shh-group MB (Figure 6).

Positive correlations in ZC4H2‒GAB1 and ZC4H2‒RNF220 were also observed (Figure 6). Furthermore, the RLIM expression level was positively correlated with GAB1, ZC4H2, and RNF220 (Figure 6). These results suggest an involvement of the RLIM‒ZC4H2‒RNF220 axis in the pathogenesis of Shh-group MB.

### Disease-causative mutations of human RLIM and ZC4H2 affect their interaction and regulation

**Figure 7:**
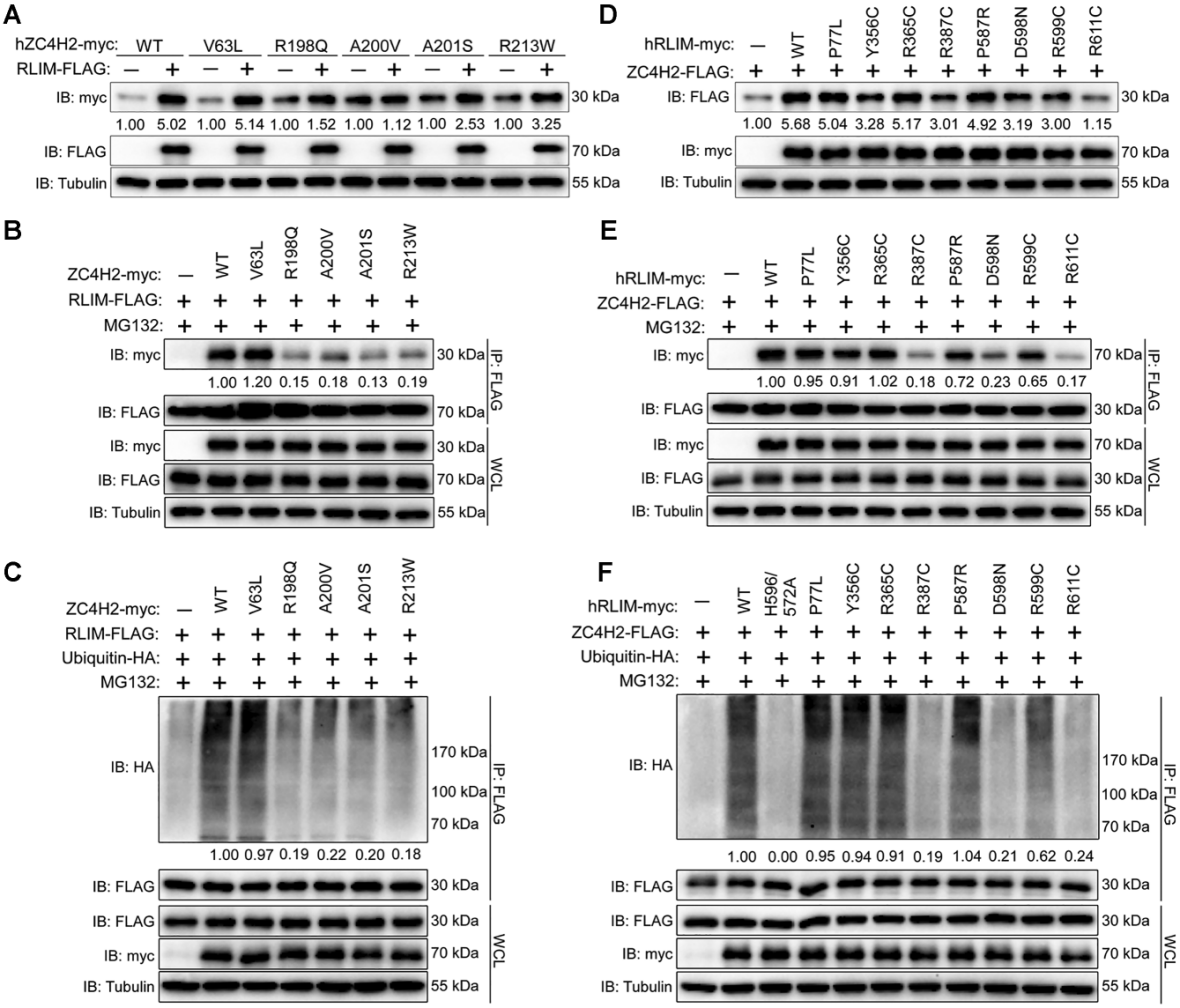
Disease-causing RLIM and ZC4H2 mutations impair their regulation. (**A**) Western blotting assays showing the effect of wild-type RLIM on protein levels of the indicated ZC4H2 mutations when coexpressed together in HEK293 cells. The ZC4H2 protein levels were normalized against α-tubulin and the statistics are shown below the indicated blot panel. (**B**) Co-IP assays showing the interaction between RLIM and ZC4H2 when the indicated ZC4H2 mutations were used. The levels of immunoprecipitated ZC4H2 protein were normalized against α-tubulin and the statistics are shown below the indicated blot panel. (**C**) *In vivo* polyubiquitination assays showing the effects of RLIM on the polyubiquitinated status of the indicated ZC4H2 mutations. The polyubiquitinated ZC4H2 protein levels were normalized against α-tubulin and the statistics are shown below the indicated blot panel. (**D**) Western blotting assays showing the effect of the indicated RLIM mutations on ZC4H2 protein levels when coexpressed together in HEK293 cells. The ZC4H2 protein levels were normalized against α-tubulin and the statistics are shown below the indicated blot panel. (**E**) Co-IP assays showing the interaction between RLIM and ZC4H2 when the indicated RLIM mutations were used. The levels of immunoprecipitated RLIM protein were normalized against α-tubulin and the statistics are shown below the indicated blot panel. (**F**) *In vivo* polyubiquitination assays showing the effects of the indicated RLIM mutations on ZC4H2. The polyubiquitinated ZC4H2 protein levels were normalized against α-tubulin and the statistics are shown below the indicated blot panel.

Mutations of both RLIM and ZC4H2 were reported to be associated with human intellectual disability (Hirata et al., 2013; May et al., 2015; Tonne et al., 2015; Frints et al., 2018, 2019). Here, we examined the effect of these mutations on their interaction and regulation. In HEK293 cells, western blotting results showed that ZC4H2 R198Q, A200V, A201S, and R213W mutations were less prone to be stabilized by RLIM (Figure 7A). Co-IP assays showed that the interaction between ZC4H2 and RLIM significantly decreased when ZC4H2 R198Q, A200V, A201S, and R213W mutations were used (Figure 7B). Additionally, less ZC4H2 was polyubiquitinated by RLIM when ZC4H2 R198Q, A200V, A201S, and R213W mutations were tested (Figure 7C). Next, we examined the effects of RLIM mutations. RLIM R387C, D598N, and R611C significantly decreased the ZC4H2-stabilizing ability, while RLIM P77L, R365C, P587R, and R599C showed weak or no reduction (Figure 7D). Co-IP results proved that the interaction between ZC4H2 and RLIM R387C, D598N, and R611C decreased, while RLIM P77L, R365C, P587R, and R599C still showed robust ability to interact with ZC4H2 in HEK293 cells (Figure 7E). Furthermore, less ZC4H2 was polyubiquitinated when RLIM R387C, D598N, or R611C was coexpressed (Figure 7F). Together, these results showed that the ZC4H2‒RLIM interaction and regulation might be involved in the pathogenesis of XLID caused by ZC4H2 or RLIM mutations.

## Discussion

In humans, a cohort of ZC4H2 mutations have been reported to be associated with X-linked neurodevelopment disorders, while the underlying mechanism of the action remains unclear (Hirata et al., 2013; May et al., 2015; Frints et al., 2019). Our previous study reported that ZC4H2 acts as an RNF220 stabilizer and that ZC4H2 knockout mice are neonatally lethal with ventral spinal cord patterning defects through RNF220-mediated fine-tuning of the Shh gradient (Ma et al., 2020b). RNF220 acts as a positive regulator of Shh signaling via an epigenetic mechanism during cerebellar development and its overexpression is associated with Shh-group MB progression (Ma et al., 2020a). In this study, we report the involvement of ZC4H2 in cerebellar development and Shh-group MB progression through RNF220-mediated Shh signaling enhancement, implying ZC4H2 as a new potential drug target for Shh-group MB treatment. Collectively, our reports demonstrate that ZC4H2 or RNF220 knockout animals phenocopy each other during many neural developmental processes, including LC and cerebellar and spinal cord development.

The RNF220/ZC4H2 protein complex is stabilized at the posttranscriptional level, as both of their proteins are extremely upregulated, while their mRNAs move toward the other side in the Shh-group MB (Figure 2I; Supplementary Figure S1B; Ma et al., 2020a). Protein ubiquitination involves the covalent attachment of the 76-amino acid ubiquitin peptide to the epsilon amino group of target lysine residues. Polyubiquitin chains can be formed by sequential addition of ubiquitin molecules through any of its seven lysines (K6, K11, K27, K29, K33, K48, and K63) or the free amino group of the initial methionine. K48-linked polyubiquitination has been extensively characterized as the canonical signal for proteasomal degradation, while the others show diverse and mostly nonproteolytic roles (Husnjak and Dikic, 2012). We show evidence here that ZC4H2 interacts with and is stabilized by the ubiquitin E3 ligase RLIM in a ligase-dependent manner (Figure 3). Although ZC4H2 also interacts with RNF220, we failed to observe the interaction between RNF220 and RLIM even in the presence of ZC4H2 by co-IP assays (data not shown). It might be the fact that RLIM and RNF220 interact with ZC4H2 in an exclusive manner. Indeed, we provided evidence that RLIM is able to induce the polyubiquitination of ZC4H2 with both K11 and K63 linkages. The K63-linked ubiquitin chain is the second most abundant ubiquitin signal in mammalian cells. K63 ubiquitination of proteins is involved in DNA repair, kinase activation, membrane protein internalization, subcellular localization, intracellular vesicular trafficking, and signaling protein complex assembly (Lim and Lim, 2011). Our data identified a function for K11/K63 polyubiquitination in protein stabilization. K11/K63 polyubiquitination may counteract the binding of other protein modifiers involved in protein degradation or favor the recruitment of protein chaperones. The mechanisms, including the structural basis of such regulation, remain to be investigated.

Importantly, Shh signaling also induces RLIM expression through transactivation by Gli, which therefore forms a reciprocal feedback loop. In addition, our study shows that depletion of RLIM inhibits MB cell growth *in vitro* and tumor formation *in vivo*, at least partially, by stabilizing the ZC4H2/RNF220 complex (Figure 5). In human Shh-group MB specimens, the expression of RLIM correlated with that of ZC4H2 and RNF220 (Figure 6). Our findings demonstrate a critical role of the RLIM‒ZC4H2/RNF220‒Shh signaling axis in Shh-group MB progression. Similar to RNF220 and ZC4H2, RLIM is also highly expressed in CGNP during cerebellum development and is required for CGNP cell proliferation *in vitro* (Figure 1; Supplementary Figure S8; Hayes et al., 2001; Ma et al., 2020a). RLIM knockout female mouse embryos died preimplantation due to insufficient X inactivation, while RLIM knockout male mice were viable and fertile (Shin et al., 2010). A conditional RLIM knockout mouse model is needed to study its potential role during cerebellum development.

RLIM has been reported to be involved in cancer development with diverse functions through targeting different proteins. In lung tumorigenesis, RLIM is involved in p53 regulation through the TRIM28‒RLIM‒MDM2 axis and restrains cell proliferation by targeting c-myc (Gao et al., 2016; Jin et al., 2020). It was also reported that RLIM promotes cell migration in osteosarcoma cells by regulating TGF-β signaling (Huang et al., 2011). In addition, RLIM suppresses hepatocellular carcinogenesis by upregulating p15 and p21 (Huang et al., 2017). Here, we provided evidence that RLIM is a direct target of Shh/Gli signaling and required for full activation of Shh signaling in MB cells. In human clinical samples, high RLIM levels are positively associated with GAB1, a Shh-group MB marker. Our data suggest that RLIM absolutely acts as an oncogene during Shh-group MB progression.

Although RLIM XLID mutations were reported to disrupt E3 ligase activity and RLIM catalytic function regulates neural stem cell differentiation (Bustos et al., 2018), whether RLIM XLID mutants affect the processing of substrates that function in neural development is unknown. We found that serial ZC4H2 and RLIM mutations isolated in patients with intellectual disorders showed defective effects on their interactions and RLIM-mediated ZC4H2 polyubiquitination (Figure 7). Although there are caveats associated with our preliminary data on the impact of RLIM XLID variants on ZC4H2 regulation, our data here indeed provided a potential mechanism by which ZC4H2 and RLIM mutations are involved in human neural disorders. Structural studies of RLIM and ZC4H2 for sure would help in this direction.

## Materials and methods

### Animals

All mice were maintained and handled according to the guidelines approved by the Animal Care and Use Committee of the Kunming Institute of Zoology, Chinese Academy of Sciences. All mice were maintained on a C57BL/6 background. The following mouse lines were used as previously reported: *ZC4H2^fl/fl^;Rosa26-CreER* and *Ptch1^+/^*^–^ (Ma et al., 2020a, b).

### Plasmids, reagents, cell lines, lentiviral preparation, infection, stable cell line construction, MTS assay, and xenograft mouse model

ZC4H2 and ubiquitin expression constructs were described previously (Ma et al., 2019, 2020b). Human and mouse RLIM plasmids were obtained from OriGene (RC203442 for human RLIM and RC209309 for mouse RLIM) and then subcloned into the PCS^2+^-FLAG or myc expression vectors. A catalytically inactive RLIM mutant (H569A/C572A) was prepared by PCR-driven overlap extension using *Pfu* DNA polymerase (Fermantas).

Cyclopamine (S1146, Selleck; 50 nM), LDE-225 (S2151, Selleck; 5 nM), GDC-0449 (S1082, Selleck; 5 nM), SAG (S7779, Selleck; 5 nM), and Shh (Z03067, GeneScripts; 500 ng/ml) were used to modulate Shh signaling in CGNP and MEF cells.

HEK293, HEK293T, Shh-N HEK293T, and Daoy cells were maintained as previously reported (Ma et al., 2020a). Primary CGNP cultures were derived from dissociated P7 mouse cerebella as previously reported (Miyazawa et al., 2000; Yue et al., 2014). Briefly, cerebella from P7 mice were cut into three pieces and incubated at 37°C for 10 min in digestion buffer containing 1 mg/ml papain and 5 U/ml DNase I (Roche). Then, tissues were triturated with pipettes to obtain a single-cell suspension. The cells were washed once with phosphate-buffered saline (PBS), loaded onto a step gradient of 35% and 65% Percoll, and centrifuged at 3000 rpm for 20 min. The cells at the 35% and 65% Percoll interface were collected, washed once with PBS, and then resuspended in Dulbecco's modified Eagle's medium (DMEM)/F12 medium. The cells were further purified by discarding adherent cells with two consecutive incubations in tissue culture dishes. Finally, the resulting CGNP cells were resuspended in DMEM/F12 medium containing 25 mM KCl, N_2_ supplements, and 10% fetal bovine serum at a density of 4 × 10^6^/ml, seeded in Lab-Tek chambered slides or cell culture plates coated with 50 µg/ml poly-D-lysine, and incubated at 37°C with 5% CO_2_.

For Shh stimulation or inhibition, Shh-N-conditioned medium, SAG, or cyclopamine was used as previously reported (Jiang et al., 2015; Ma et al., 2020a). All cells were cultured in a humidified incubator with 5% CO_2_ at 37°C. *ZC4H2^fl/^^y^;Rosa26-CreER* CGNPs were treated with 2 ng/ml 4-OHT for 3 days to induce ZC4H2 knockout.

shRNA sequences targeting human ZC4H2-1# (5′-GAGGCTGCATGATGAGTATAA-3′), ZC4H2-2# (5′-GCCCATATCTTGTTTCAGGTA-3′), RLIM-1# (5′-GAGCTATCAGTCAGCCTTTAC-3′), RLIM-2# (5′-GCGTACACACACACACACATTTA-3′), mouse RLIM-1# (5′-ATCAATTTGTGAATAATCTCA-3′), and RLIM-2# (5′-TACTGAGGAAGAGTTGCTCAG-3′) were cloned into the pLKO.1 lentiviral vector. Lentiviral preparation, infection, stable cell line construction, and Dox-induced knockdown were conducted as previously reported (Ma et al., 2020a).

MTS assay and Daoy cell xenograft mouse model were conducted as previously reported (Ma et al., 2020a).

### IP, *in vivo*/*in vitro* ubiquitination, and western blotting assays

IP, *in vivo* ubiquitination, and western blotting assays were conducted as previously reported (Ma et al., 2014). For IP, cells or tissues were homogenized and lysed in IP lysis buffer (50 mM Tris-HCl [pH 7.4], 150 mM NaCl, 5 mM EDTA [pH 8.0], and 1% Triton X-100) that contained a protease inhibitor mixture (Roche Applied Science) for 30 min on ice. Following centrifugation at 14000 rpm for 15 min at 4°C, 90% of the supernatant was incubated with FLAG-M2 beads or RLIM antibody (Sigma) at 4°C for 4 h. The bound proteins were eluted with sodium dodecyl sulphate (SDS) loading buffer at 95°C for 5 min. For *in vivo* ubiquitination, transfected HEK293 cells were treated with MG132 at 25 nM for 6 h prior to harvesting. Harvested cells were lysed in SDS denaturing lysis buffer (50 mM Tris-HCl [pH 6.8], 1.5% SDS) at 95°C for 15 min. Following 10-fold dilution of the lysate with extraction buffer C–bovine serum albumin (BSA) (50 mM Tris-HCl [pH 6.8], 180 mM NaCl, 0.5% NP-40, and 0.5% BSA) plus protease inhibitors (Roche), the cell lysates were immunoprecipitated with anti-FLAG M2 beads (Sigma). The bound proteins were eluted with Laemmli sample buffer at 95°C for 5 min. Total lysates and immunoprecipitates were subjected to SDS–polyacrylamide gel electrophoresis and western blotting analysis.

*In vitro* ubiquitination assays were carried out as previously reported (Ma et al., 2019). Briefly, the assays were performed in a 50 µl reaction volume containing the following components: 5 µg WT, K63, or K11 ubiquitin (Boston Biochem), 0.2 µg E1 (Boston Biochem), 0.2 µg E2 (UbcH5h, Boston Biochem), 1 µg streptavidin-purified RLIM-myc, 1 µg ZC4H2-FLAG protein (purified with anti-FLAG beads), 5 µl 10× reaction buffer (Boston Biochem), 2 mM ATP (Cell Signaling), and 5 mM MgCl_2_ (Sigma). Reactions were incubated at 30°C for 1 h, terminated by boiling with SDS loading buffer, and then processed for western blotting with the indicated antibodies.

The following primary antibodies were used: anti-Gli1 (2553S, Cell Signaling Technology), anti-Ptch1 (17520-1-AP, Proteintech), anti-Hhip1 (11654-1-AP, Thermo Fisher Scientific), anti-RNF220 (HPA027577, Sigma-Aldrich), anti-ZC4H2 (HPA049584, Sigma-Aldrich), anti-α-tubulin (66031-1-Ig, Proteintech), anti-RLIM (16121-1-AP, Thermo Fisher Scientific), anti-FLAG (F7425, Sigma-Aldrich), anti-myc (C3956, Sigma-Aldrich), and anti-ubiquitin (sc-8017, Santa Cruz Biotechnology).

### RNA isolation, reverse transcription, and RT-PCR

Total RNA isolation, reverse transcription, and RT-PCR were conducted as previously reported (Ma et al., 2020a). The following primers were used: mouse actin: 5′-GCCAACCGTGAAAAGATGAC-3′ and 5′-GAGGCATACAGGGACAGCAC-3′; mouse ZC4H2: 5′-AGCAGGACACAAGGCAGACA-3′ and 5′-TTGCAAAGAGGGCATATAGG-3′; mouse RNF220: 5′-GTCTCAGTAGACAAGGACGTTCACA-3′ and 5′-GGGGTGGAGGTGTAGTAAGGAAG-3′; mouse RLIM: 5′-GGTCCACCACCACAGAGC-3′ and 5′-TGACCACTTCTTGTTGTATTTCC-3′; mouse Gli1: 5′-GGAGACAGCATGGCTCACTA-3′ and 5′-GAGGTTGGGATGAAGAAGCA-3′; mouse Ptch1: 5′-CATGGCCGCATTGATCCCTA-3′ and 5′-GGGTGTTACTGTGAGGCTCTG-3′; mouse Hhip1: 5′-GGAGTAACCCTCACTTCAACAGCA-3′ and 5′-CCCGTTGGAATCTGAGCAAAGTA-3′; human actin: 5′-ACCGAGCGCGGCTACAG-3′ and 5′-CTTAATGTCACGCACGATTTCC-3′; human RLIM: 5′-GTAGGTCACCTCTGCATCCA-3′ and 5′-GAACTTCCCTCCGTCTCATT-3′; human RNF220: 5′-GATGCCATCCACCAGCAA-3′ and 5′-CACGAGATAGCTGCCGTTCA-3′; human ZC4H2: 5′-AATGACCTAAACAAGCTGCT-3′ and 5′-TTCTCCTCTTCTTCACACAA-3′; human Gli1: 5′-TGTGTATGAAACTGACTGCCG-3′ and 5′-CCCAGTGGCACACGAACTC-3′; human Ptch1: 5′-TGTGCGCTGTCTTCCTTCTG-3′ and 5′-CACGGCACTGAGCTTGATTC-3′; and human Hhip1: 5′-ATTGCTTCCCTAATGTCCT-3′ and 5′-GGGAGGTAGACCCACACCA-3′.

### ChIP‒qPCR assays

ChIP was conducted as previously reported (Ma et al., 2020a) with anti-Gli1 (2553S, Cell Signaling Technology) and anti-IgG (2729S, Cell Signaling Technology). Input or precipitated genomic DNA was used as a template for RT-PCR with the following primers: mouse RLIM GBS1: 5′-TTAGAGACATTGCCTAATTTTC-3′ and 5′-CAAGGATTAAGAGTAAGTACTTC-3′; mouse RLIM GBS2: 5′-CAGCCAATTTCTAACAAATTG-3′ and 5′-TTCAGAGTCAGTTCCTCAGTAG-3′; human RLIM GBS1: 5′-GTGACATTTTGAGCTATTCCACAG-3′ and 5′-CATAATGCTAGTCCTAGATTAATTG-3′; and human RLIM GBS3: 5′-CTCAGCGATATTTTAGACTTCAG-3′ and 5′-CAGGAGATAAATGTGTATTCTGC-3′.

### Immunofluorescence staining and BrdU incorporation assay

Immunofluorescence staining and BrdU incorporation assay on cultured Daoy cells or tissue sections were performed as previously described (Ma et al., 2020a). The following primary antibodies were used: anti-RLIM (16121-1-AP, Thermo Fisher Scientific); anti-ZC4H2 (HPA049584, Sigma-Aldrich); anti-RNF220 (HPA027577, Sigma-Aldrich); anti-BrdU (RF06, Bio-Rad); anti-Ki67 (ab15580, Abcam); and anti-NeuN (MAB377, Sigma-Aldrich).

### Tissue sectioning and immunohistochemical staining

Tissue sectioning and immunohistochemical staining were performed as previously described (Ma et al., 2019, 2020a). Immunohistochemistry on clinical MB tissue array samples (Bra01022 and BC17012b, Alenabio) was performed. There were 36 independent human MB cases with two samples for each case on the slide. The array samples were subjected to GAB1 staining to distinguish Shh and other subgroups of MB. Anti-RLIM (16121-1-AP, Thermo Fisher Scientific), anti-RNF220 (HPA027577, Sigma-Aldrich), anti-ZC4H2 (HPA049584, Sigma-Aldrich), and anti-GAB1 (720334, Invitrogen) were used. Chromogenic development was performed with a DAB detection kit (Beyotime Biotechnology) according to the manufacturer's instructions. The percentage of positive cells and staining intensity were multiplied to produce a weighted score for each case.

### Statistical analysis

Each experiment was conducted at least three times with the same results. Data were analyzed using GraphPad software as follows: for experiments including RT-PCR, MTS assay, and BrdU staining, statistical significance was evaluated using the two-tailed Student's *t*-test for comparison of two groups; for associations between gene expression values, significance was evaluated by Pearson product moment correlation coefficient analysis. ImageJ software was used to quantify the western blotting data. An Olympus IX73 microscope and CellSens software were used for cerebellum size quantification. The cerebellum EGL length was normalized to body weight.

## Supplementary Material

mjab082_Supplemental_File

## Notes

**Author contributions:** conceptualization: Yan Li, P.M., and B.M.; methodology: P.M., Yuwei Li, C.Y., Q.K., H.W., L.Z., L.L., and Y.C.; formal analysis: P.M. and Yan Li; investigation: P.M., Yuwei Li, C.Y., H.W., and Y.C.; writing original draft: P.M. and Yuwei Li; revising and editing: B.M. and Yuwei Li; supervision: B.M.; project administration: P.M., S.Z., and B.M.; and funding acquisition: P.M., S.Z., Yan Li, and B.M.
