# Supplementary material for: Sequential stabilization of RNF220 by RLIM and ZC4H2 during cerebellum development and Shh-group medulloblastoma progression
Source: J Mol Cell Biol. 2022 Jan 18;14(1):mjab082. doi: 10.1093/jmcb/mjab082 (PMC8982406; doi:10.1093/jmcb/mjab082)
Supplement: mjab082_Supplemental_File [file mjab082_supplemental_file.pdf]

Supplementary material

Supplementary Figure S1 (related to Figure 1)

Supplementary Figure S2 (related to Figures 1 and 2)

Supplementary Figure S3 (related to Figure 3)

Supplementary Figure S4 (related to Figure 3)Supplementary Figure S5 (related to Figure 3)

Supplementary Figure S6 (related to Figure 4)

Supplementary Figure S7 (related to Figure 4)

Supplementary Figure S8 (related to Figure 5)

Supplementary Figure S9 (related to Figure 5)

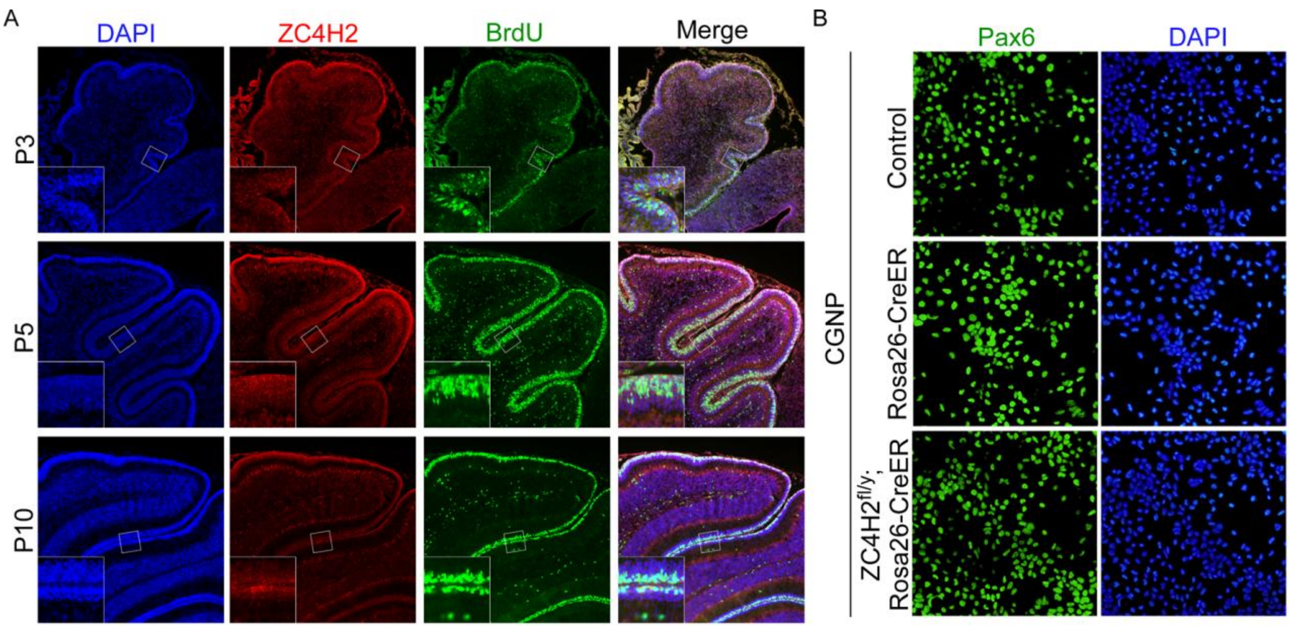

Supplementary Figure S1 (related to Figure 1) (**A**) ZC4H2 (red) and BrdU (green) co-staining immunofluorescence assay in cerebellum at different developmental stages. Scale bar: 200 μm. (**B**) Immunofluorescence staining showing the the expression of Pax6 in isolated CGNPs from the indicated mice.

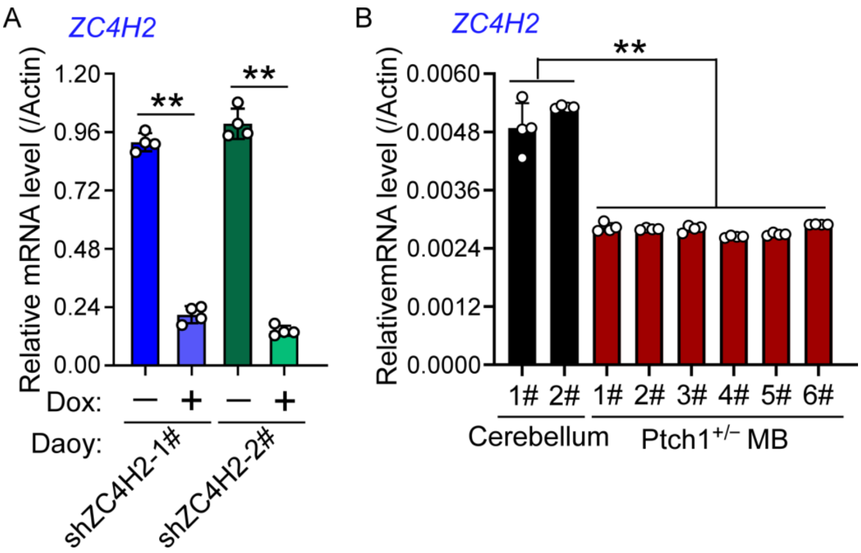

Supplementary Figure S2 (related to Figures 1 and 2) (**A**) RT-PCR assays showing the relative mRNA level of ZC4H2 in shZC4H2-1# or shZC4H2-2# stably transfected Daoy cell in the presence of Dox or not. (**B**) RT-PCR assays showing the relative mRNA level of ZC4H2 in control cerebellum or *Ptch1*$^{+/-}$ medulloblastoma tissues. β-Actin was used as a loading control. ***P* < 0.01 (Student's *t*-test). MB, medulloblastoma.

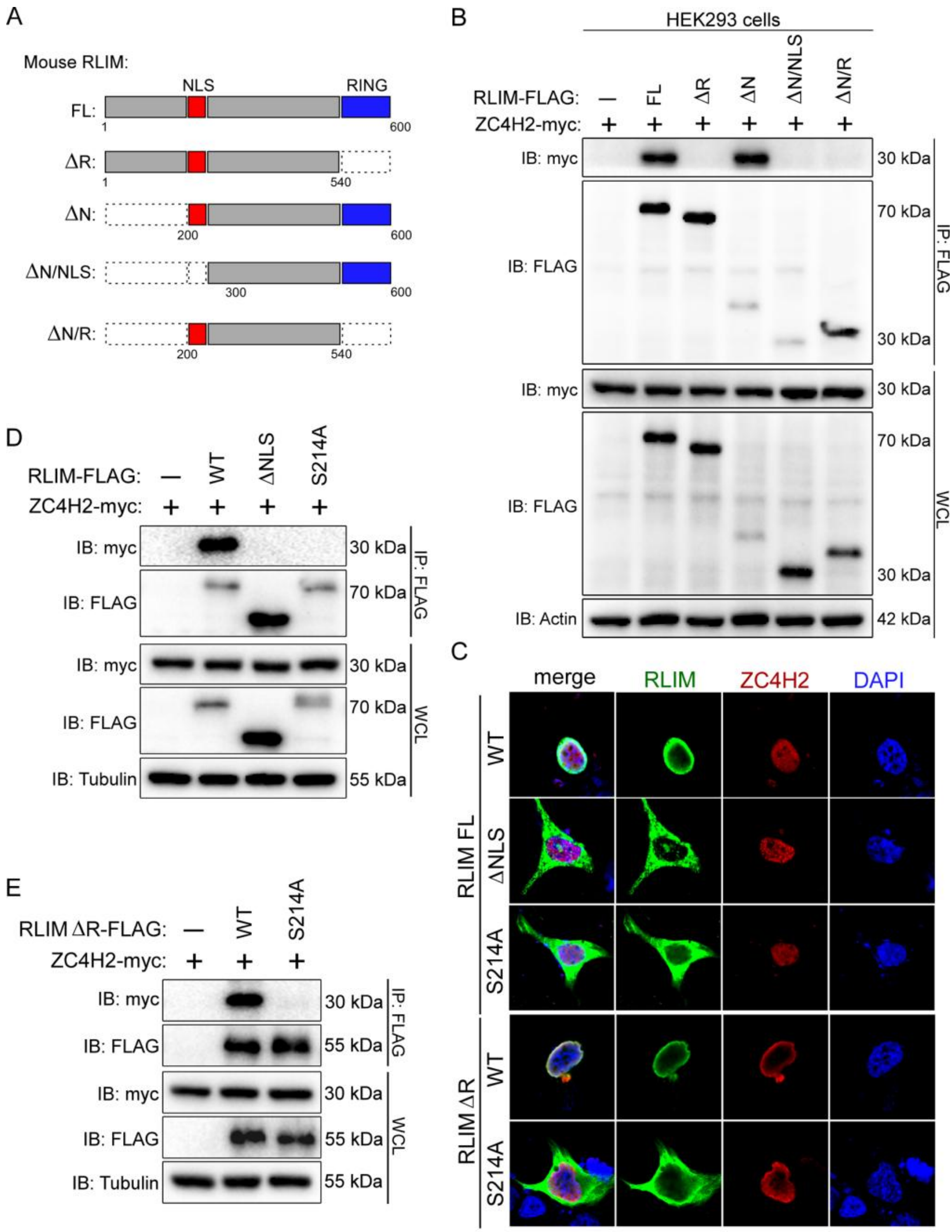

Supplementary Figure S3 (related to Figure 3) RLIM interacts with ZC4H2. (**A**) A table summarizing Y2H results using ZC4H2 as a biat. (**B**) Schematic representation of mouse RLIM truncates used in co-IP assays. FL, full-length. NLS, nuclear localization signal. ΔR, RING domain deletion. ΔN, N terminal 1–200 aa deletion. ΔN/NLS, both N terminal 1–200 aa and nuclear

localization signal deletion. ΔN/R, N terminal 1–200 aa and RING domain deletion. (**C**) co-IP assays showing the interaction between ZC4H2 and RLIM when different RLIM truncates were used. IB, immunoblot. IP, immunoprecipitation. WCL, whole-cell lysate. (**D**) Co-staining of immunofluorescence assays showing the localization of ZC4H2 (red) and the indicated RLIM truncates or mutations (green). WT, wild-type; ΔNLS, nuclear localization signal deletion; S214A, serine 214 mutated to alanine. (**E**, **F**) co-IP assays showing the interaction between ZC4H2 and the indicated RLIM truncates or mutations when co-expressed together in HEK293 cells.

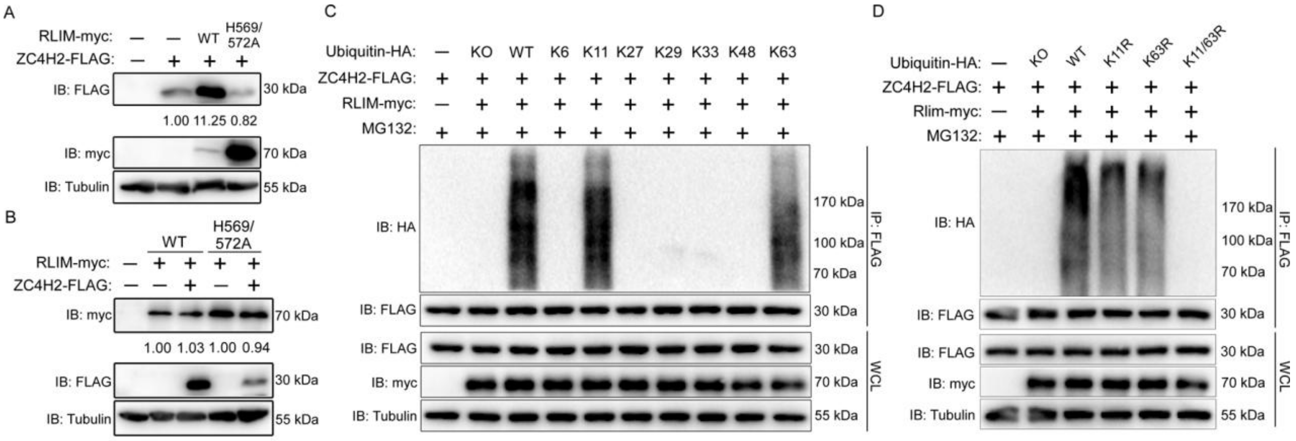

Supplementary Figure S4 (related to Figure 3) RLIM stabilizes and targets ZC4H2 for polyubiquitination. (**A**) Western blotting results showing the protein level of ZC4H2 in the presence of wild-type (WT) RLIM or its ligase dead mutation (H569/572A) in HEK293 cells. H569/572A, both histidine 569 and histidine 572 mutated to alanine. IB, immunoblot. (**B**) Western blotting result showing the effects of ZC4H2 co-expression on the protein levels of WT RLIM or its ligase dead mutation (H569/572A). (**C**) *In vivo* polyubiquitination assays showing the level of polyubiqutinated ZC4H2 in the presence of RLIM when different ubiquitin mutations were used. KO, all lysines of ubiquitin mutated to arginine; K6/11/27/29/33/48/63, all lysines except lysines 6/11/27/29/33/48/63 mutated to arginine. IP, immunoprecipitation; WCL, whole-cell lysate. (**D**) *In vivo* polyubiquitylation assays showing the ability of RLIM to ubiquitylate ZC4H2 when the indicated ubiquitin mutation constructs were used. RLIM promotes ZC4H2 for mixed polyubiquitylation of K63 and K11 lineage. K63R and K11R, lysine 63 or lysine 11 of ubiquitin mutated to arginine, respectively; K11/63R, both lysine 63 and lysine 11 of ubiquitin mutated to arginine.

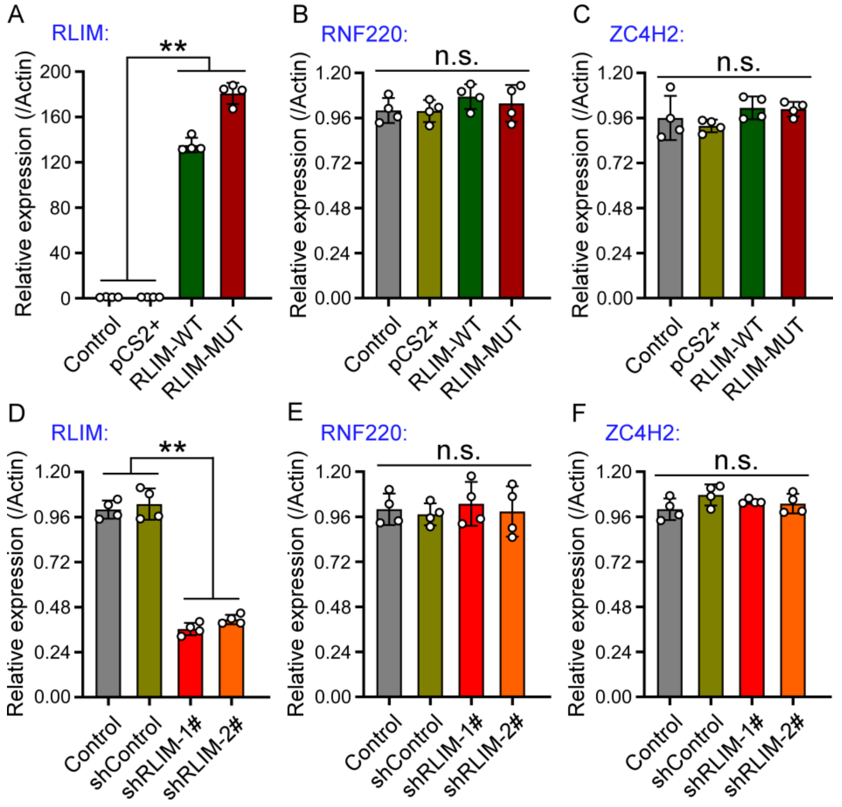

Supplementary Figure S5 (related to Figure 3) RT-PCR results showing the relative mRNA levels of RLIM, RNF220, or ZC4H2 in RLIM overexpressing HEK293 cells (**A**–**C**) or in RLIM knockdown Daoy cells (**D**–**F**). WT, wild-type; MUT, E3 ligase-dead mutation.

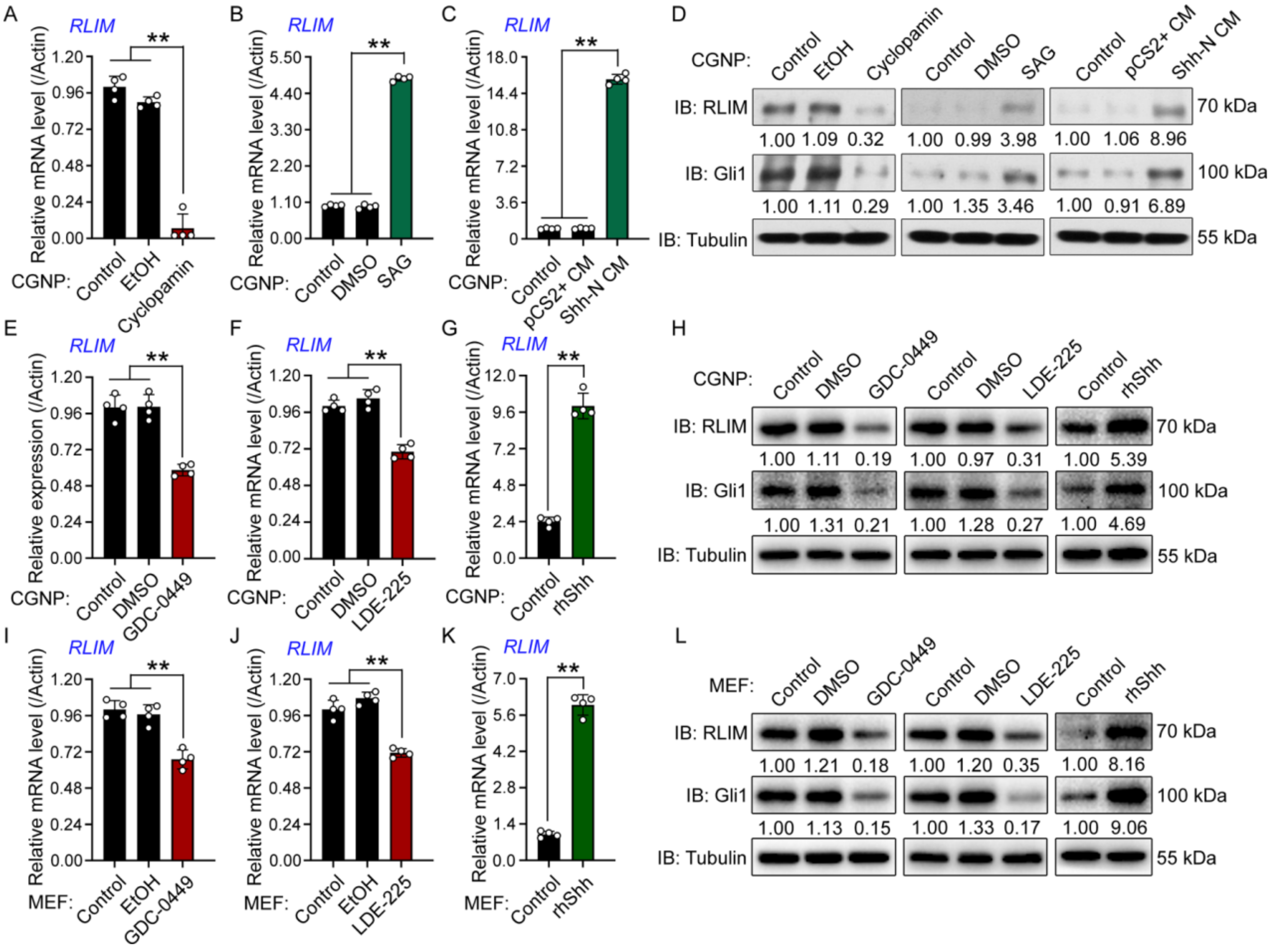

Supplementary Figure S6 (related to Figure 4) RT-PCR and western blotting results showing RLIM expression levels in CGNP or MEF cells when Shh signaling is modulated by the indicated reagents. β-Actin was used as a loading control for RT-PCR assays. The levels of indicated proteins were normalized against α-Tubulin and the statistics were labeled below each blot panel. IB, immunoblot. CM, conditioned medium.

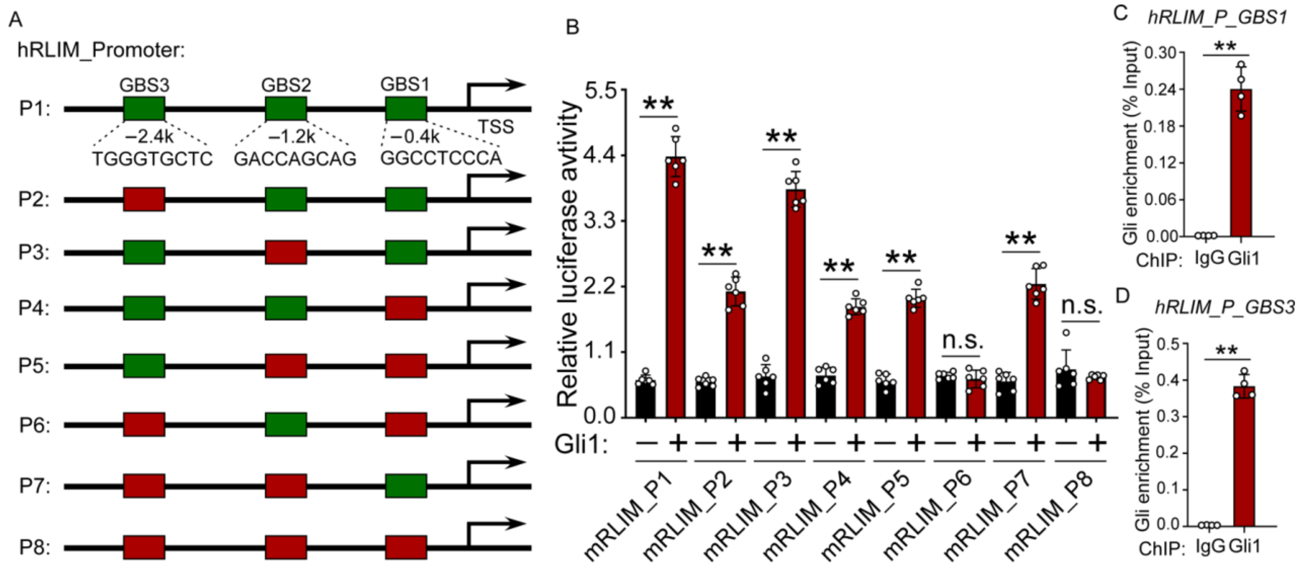

Supplementary Figure S7 (related to Figure 4) RLIM is a direct target of Gli in Daoy cells. (**A**) Schematic presentation of luciferase reporter vectors of human RLIM promoter containing the three wild-type (green) or mutated (red) predicted potential Gli-binding sites (GBSs) used in the reporter assays. TSS, transcription start site. (**B**) Luciferase reporter assays showing that two of three predicted potential GBSs are functional in human RLIM promoter. RLU, relative luciferase units. (**C**, **D**) ChIP–qPCR assays showing that Gli1 directly binds to human RLIM promoter at the two functional GBSs.

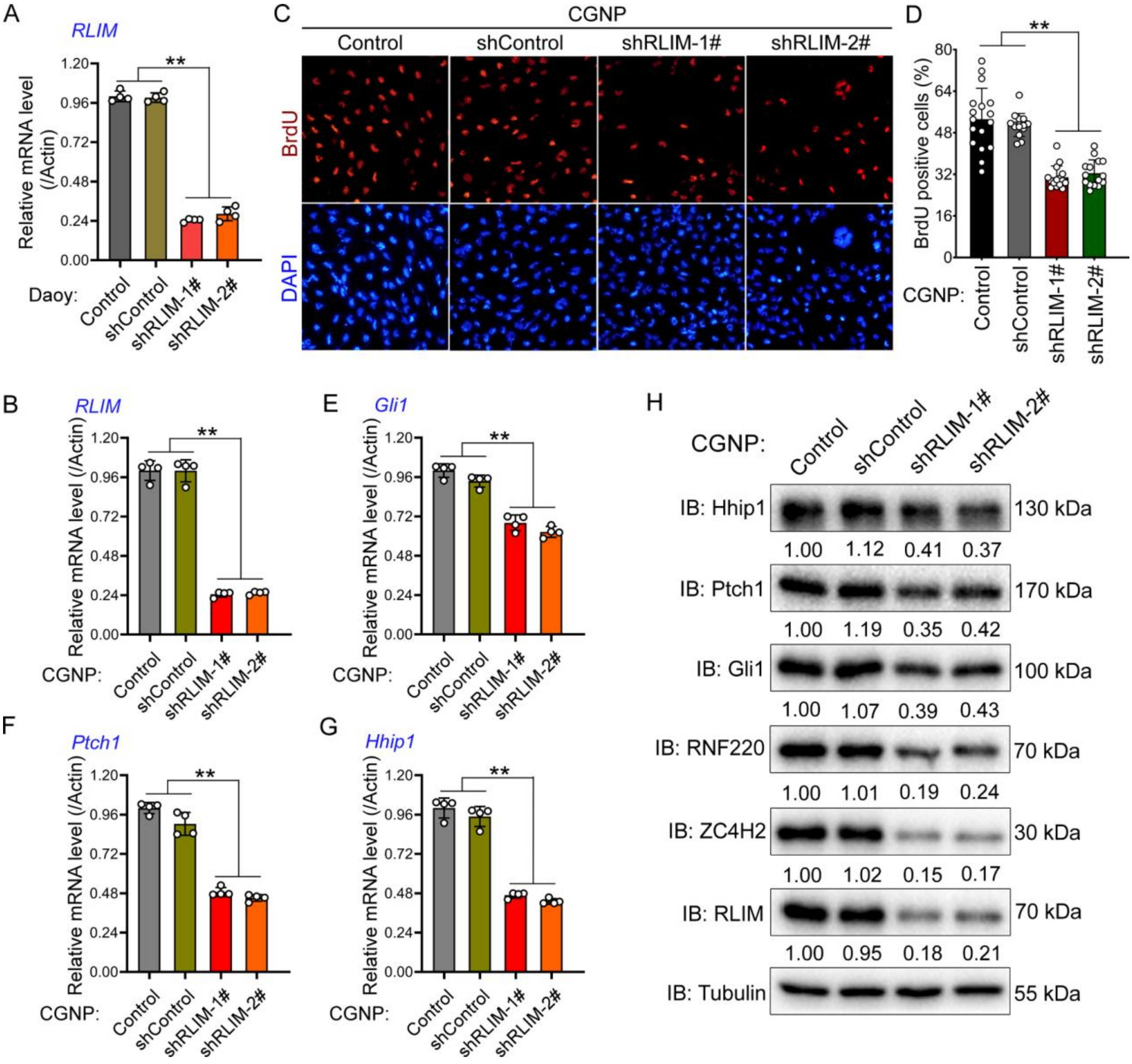

Supplementary Figure S8 (related to Figure 5) RLIM is required for proliferation and Shh signaling in CGNP cells. (**A**) RT-PCR assays showing the relative mRNA level of RLIM in the indicated Daoy cell lines. β-Actin was used as a loading control. (**B**) RLIM (red) and BrdU (green) co-staining immunofluorescence assay in cerebellum at different developmental stages. Scale bar: 170 μm (P3); 350 μm (P5) and 940 μm (P10). (**C**) RT-PCR assays showing the relative mRNA level of RLIM in CGNP cells transfected with lentivirus expressing the indicated shRNAs. (**D**, **E**) BrdU incorporation assay to evaluate DNA synthesis and proliferation rates of CGNP cells transfected with lentivirus expressing the indicated shRNAs (**D**) and the statistics were shown in (**E**). Scale bar, 50 μm. $^{**}P < 0.01$ (Student's *t*-test). (**F**–**I**) RT-PCR (**F**–**H**) and western blotting (**I**) assays showing the expression levels of Gli1 (**F**, **I**), Pthc1 (**G**, **I**), and Hhip1 (**H**, **I**) in CGNPs transfected with lentivirus expressing the indicated shRNAs. β-Actin was used as a loading control for RT-PCR assays. The levels of indicated proteins were normalized against the level of α-Tubulin and the statistics were labeled below each blot panel. IB, immunoblot.

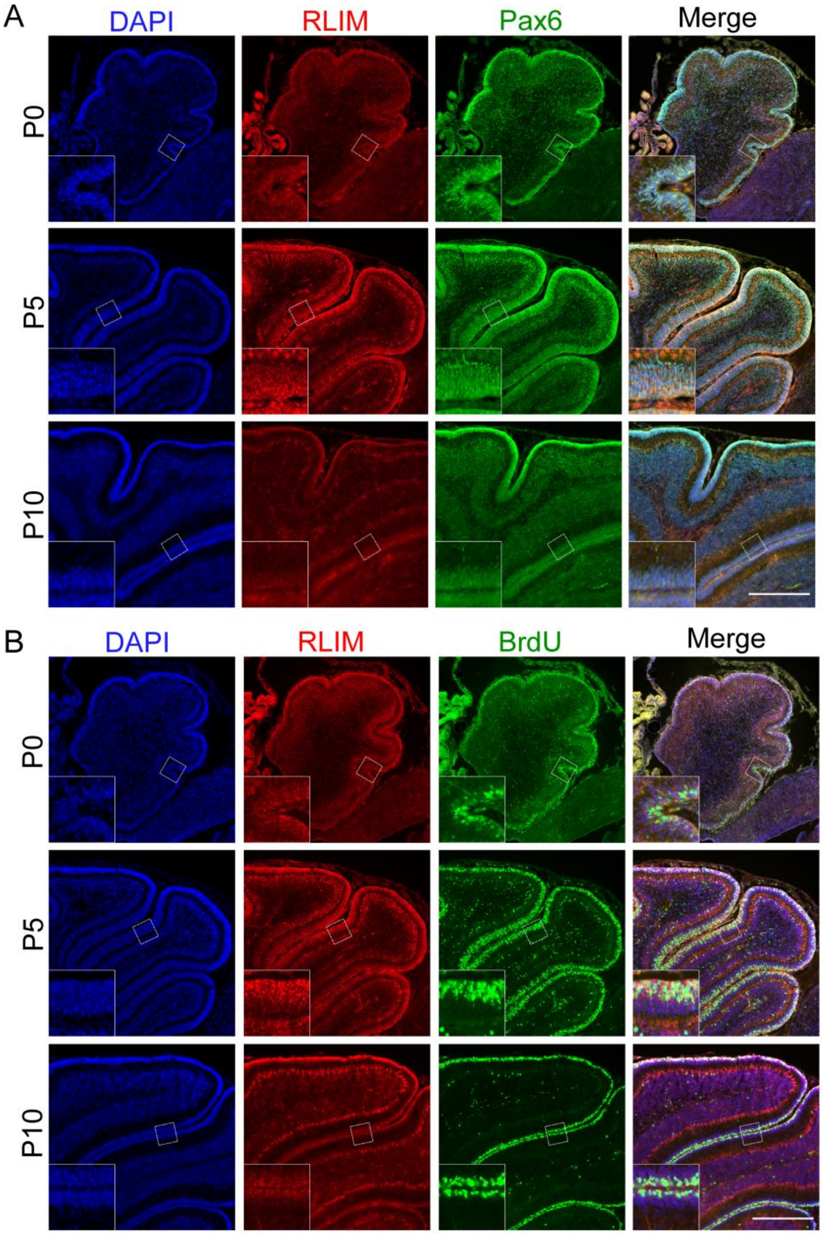

Supplementary Figure S9 (related to Figure 5) RLIM expression during developing cerebellum. RLIM (red) and Pax6 (green) (**A**) or BrdU (green) (**B**) co-staining immunofluorescence assay in cerebellum at different developmental stages. Scale bar: 200 μm.
